# Implementation of objective structured clinical examination among midwifery and nursing educators at South and Central Region Universities in Ethiopia, 2023

**DOI:** 10.1371/journal.pone.0356551

**Published:** 2026-08-26

**Authors:** Zerihun Demessie Dilebo, Bekana Fekecha Hurissa, Kebenesa Angasu Kitaba

**Affiliations:** 1 Department of Midwifery, Hossana College of Health Science, Hossana, Ethiopia; 2 Department of Midwifery, Jimma University, Jimma, Ethiopia; Ege University Faculty of Medicine, TÜRKIYE

## Abstract

**Introduction:**

The Objective Structured Clinical Examination (OSCE) is a well-known clinical skill evaluation tool that has been applied worldwide to teach and assess learners’ competencies in healthcare disciplines. Poor implementation of OSCE can lead to inaccurate assessments and ultimately result in the production of incompetent professionals. However, there is no evidence of the implementation status and factors that limit the much-needed OSCE practice in the study area.

**Objective:**

To assess OSCE implementation among midwifery and nursing educators in the South and Central region of Ethiopian universities, 2023.

**Methods:**

An institution-based cross-sectional study with a mixed approach was used among 269 midwifery and nursing educators and 15 key informants from 8 May to 10 June 2023. All midwifery and nursing educators were included from randomly selected 4 universities in southern and central Ethiopia. Quantitative data were collected using pretested self-administered questionnaires and were analyzed using descriptive statistics and logistic regression. With a p-value of less than 0.05, which denotes statistical significance, a corresponding 95% confidence interval (CI) was calculated. Qualitative data was collected through in-depth interviews and analyzed using open-code software Version 4.02, and findings were used to support quantitative findings.

**Results:**

A total of 260 (97%) educators participated in this study, and 43.5% (95% CI 37.4–49.5) of them had a good OSCE implementation. Training in OSCE (AOR = 2.07, 95% CI 1.03–4.17), having an OSCE checklist (AOR = 1.89, 95% CI 1.05–3.42), having equipment (AOR = 2.36, 95% CI 1.03–5.39) and negative perceptions toward OSCE (AOR = 0.53, 95% CI 0.31–0.92) were significantly associated, and poor organization and planning, perceptions towards OSCE, shortage of resources and insufficient number and duration of the station were found by qualitative study.

**Conclusions:**

Less than half of educators have had a good OSCE implementation. Training, equipment, checklists, and perceptions highlight issues such as poor organization and planning, resource, and time allocation stations found as influencing factors. In addition to improving in-service and ongoing OSCE training, it is preferable to ensure the availability and accessibility of resources in the facilities to improve OSCE implementation.

## Introduction

The Objective Structured Clinical Examination (OSCE) is a globally recognized, multipurpose evaluative tool designed to assess the clinical competency of healthcare professionals with a high degree of objectivity and reliability [1,2]. Since its introduction in 1975, the OSCE has been widely adopted in medical, nursing [3], and midwifery education as a robust method for teaching and evaluating clinical skills such as history-taking, physical examination, communication, and procedural competence at multiple stations within a specified time frame, and students rotate through stations, observed one-on-one by examiners using simulators [4–7].

OSCE can be used for both formative and summative purposes; it promotes active learning and critical thinking, which enhances patient safety [8,9].

It is considered a gold standard for clinical assessment due to its balance of validity, reliability, and educational impact [10]. When accompanied by feedback, it serves as both an assessment and a learning tool, leading to longer-lasting skills compared to other teaching methods, and currently available evidence confirms that OSCE is more objective, valid, and reliable than conventional exams like short- or long-case formats [11–15].

However, effective implementation requires significant resources, including trained examiners, standardized checklists, simulation equipment, and logistical coordination and management, and understanding potential issues, which are often constrained in low-resource settings, potentially compromising its fidelity and effectiveness [16–20].

In Ethiopia, the implementation of OSCE has a relatively recent history, with institutions and stakeholders working intensively from the outset to ensure its adoption [21], However, its development is not up to national expectations. even despite the recent government demand to include it in the format of a healthcare license exam and its use as both a formative and summative assessment tool within the competency-based curricula of undergraduate midwifery, nursing, and other health disciplines [17,21,22].

Existing studies within the country have primarily examined student and examiner perceptions of OSCE, generally reporting favorable attitudes but also identifying recurrent barriers such as inadequate training, scarcity of materials, poor organization, and time constraints [17,21,22]. Despite these insights, a critical evidence gap remains: there is limited empirical research assessing the actual implementation status of OSCE among educators, specifically, the extent to which they are able to correctly design, administer, and evaluate OSCE in line with national guidelines. Without a clear understanding of educators’ implementation practices and the contextual determinants influencing them, efforts to enhance clinical assessment systems may lack direction and fail to address the root causes of poor OSCE execution.

This study therefore aimed to assess the implementation status of OSCE among midwifery and nursing educators in universities in the South and Central regions of Ethiopia and to identify the factors associated with its application. By employing a concurrent mixed-methods design, the study not only quantifies the level of OSCE implementation but also qualitatively explores the lived experiences, perceived challenges, and systemic barriers faced by educators and students. The findings are intended to provide actionable evidence to academic institutions, curriculum planners, and policymakers to inform targeted interventions that can enhance the quality, consistency, and educational impact of OSCE.

Also, this study offers a comprehensive analysis of OSCE implementation in low- and middle-income countries, providing valuable insights to the existing body of knowledge. It uniquely highlights the distinct socio-political and economic factors that influence OSCE execution in these settings, while emphasizing the critical material, human, and financial elements that affect its success. By addressing the gap in the Ethiopian context specifically, this research lays a foundation for future studies and policy-making in the region, which is essential for producing competent health professionals to serve the community.

## Methods

### Setting and design of the study

This study was carried out in the South and Central regions of the Ethiopian University School of Midwifery and Nursing. Currently, there are six public universities in the south and central regions regulated by the Federal Ministry of Education. Arba Minch, Wolaita Sodo, Wachemo, Worabe, Dila, and Wolkite Universities.

The distance from Addis Ababa to these institutions is approximately 430 km, 317 km, 230 km, 176 km, 351, and 166 km, respectively. The study was carried out between 8 May and 10 June 2023.

An institution-based cross-sectional study was used that uses quantitative data supported by a qualitative study. Which integrates both quantitative and qualitative data to provide a comprehensive understanding of the research problem. This approach allows for triangulation of data, enhancing the validity of the findings. The study followed a pragmatic research paradigm, using mixed methods to gain a comprehensive understanding of OSCE implementation practices.

All midwifery and nursing educators, midwifery and nursing students who were third year or above from Ethiopian universities in the south and central regions were the source population. Participants who were recruited as regular academic staff from south and central Ethiopian universities with >6 months of work experience and who have a BSc degree and above were included.

The sample size and sampling methods were carefully designed to collect quantitative and qualitative data effectively. For the quantitative aspect, four universities (i.e., Wachemo, Wolkite, Arba Minch, and Wolaita Sodo universities) were randomly selected out of the six universities under study in the south and central regions. Interestingly, all 269 midwifery and nursing educators from these universities were included in the research sample. Given the relatively small number of study participants, opting for a census method was practical and ensured comprehensive coverage [23].

However, for qualitative data collection, a purpose-sampling method was used. This involved the deliberate selection of 15 key informants (KI) based on their years of experience and their positions within the school. Additionally, specifically, to gain a more nuanced perspective, class representatives and students in their third year or higher were selected for in-depth interviews.

The determination of the qualitative sample size selected for this study was based on the principle of data saturation. According to recent evidence, our definition of data saturation refers to the unnecessary collection of additional data based on what has already been collected; it signifies the number of interviews needed until no new information is evident (no new themes emerged from additional interviews, and repetition of codes) and aligns with the research question(s). Accordingly, based on our research question and method, we confirmed the adequacy of the data, indicating that data saturation occurs among fifteen key informants. Data redundancy began to appear after thirteen participants. The number of participants interviewed continued up to fifteen, establishing the sample size based on the richness of the information or the saturation of the data achieved. This process guided the final sample size of fifteen, ensuring comprehensive coverage without unnecessary redundancy.

### Data collection and procedure

The questionnaire was developed after reviewing different types of literature and making it fit the objectives of the study [7,17,18,20]– [22,24]. Tools were pre-tested among 26 educators and students who were not a part of the main study. The tools had five sections: sociodemographic, facility-related characteristics, perception toward OSCE, and OSCE implementation. Two semi-structured in-depth interview guides were used (one for educators and the other for students), and they contained different questions.

For quantitative data collection, a pre-tested self-administered questionnaire was used. Data were collected by four data collectors (BSc Midwife) and four supervisors (Master of Science degree in public health), i.e., one data collector and one supervisor for each university. We chose them based on their experience with data collection. Then they were trained on the objectives of the study, the survey, and the data collection process. Principal investigators and supervisors double-checked the surveys to ensure they were complete and relevant. They agree to collaborate closely to ensure that all survey tools are, all necessary questions are included. The survey flow is logical and easy for participants to understand and the questions directly address the research objectives and accurately capture the intended information.

Qualitative data collection, In-depth interviews were conducted to collect data through face-to-face interactions. All interviews were conducted in separate locations where others were unable to listen to the interviews. The interviews lasted between 60 and 80 minutes. Data were recorded audio with the permission of study participants, and field notes (handwritten) were taken to take non-recordable gestures. Probes were used to obtain detailed information from the respondents whenever necessary. The interviews continued until the data saturation level.

### Quality control

Before the actual data collection; a pre-test was done with 10% of midwifery and nursing educators and students in the central region, of Hosanna Health Science College. Based on the findings from the pretest, some ambiguous questions and logical order were corrected.

An ongoing formative checkup for completeness and consistency of responses was made by the data collectors daily. Data cleaning and reviewing for mistakes, inconsistencies, and adjustments were conducted to avoid errors in the labeling or order of the variables of interest. The collected data were reviewed, checked, coded, and cleaned for completeness.

Internal consistency (reliability) was tested by calculating Cronbach’s Alpha using SPSS version 26. The overall standardized Cronbach’s alpha for internal consistency or reliability score of OSCE implementation and perception measurement was 0.796 and 0.721, respectively. These values demonstrate the acceptable internal consistency of our measurement instruments.

Additionally, health behavior experts cross-checked the validity of the tool’s content. Data collectors and supervisors were trained in the study instrument and data collection procedure. The principal investigator and the supervisors checked the completeness of the collected data.

In this study, the credibility of the qualitative study was ensured using the triangulation of data, transferability was ensured by a clear and thick description of the study, and the interview guide was developed without being influenced or dependent extensively on literature review evidence, dependability through conducting peer debriefings among data collectors to get their alternative explanations and similar consensus on emerging codes and to evaluate whether or not the findings are supported by data. Conformability was ensured through bracketing (that is, the interpretations of the data were derived from the collected data and not based on the research’s viewpoints).

### Measurement and operational definitions of the variables

The implementation of OSCE was evaluated using the questionnaire developed from an OSCE implementation guide of the Ethiopian Ministry of Health. This questionnaire consisted of 11 items with 5-point Likert scale items. Educators were asked to rate on a five-point scale (1 = strongly disagree, 2 = disagree, 3 = neutral, 4 = agree, 5 = strongly agree), how they implement the OSCE.

The overall OSCE implementation score (new variable) was calculated from the sum of the responses of the respondents for each item related to the implementation. The composite variable ‘implementation status’ was then dichotomized using the median score as the cutoff point. The median score was 33. Thus, respondents who scored the median score or higher were classified as having a good OSCE implementation and otherwise poor [7]. Composite scores were dichotomized, and logistic regression was used to identify predictors of good implementation.

Perception towards OSCE implementation has 11 items that were measured by using a Likert scale where educators were supposed to strongly agree, agree, neutral, disagree, and strongly disagree [24]. The overall perception score (new variable) was calculated from the sum of the responses of the respondents for each item related to perception. Then the perceptions of the OSCE of the respondents were dichotomized using the median score as the cutoff point. The median score was 42. The perceptions of the respondents were considered ‘positive’ if the cut-off point was equal to the median score and above and ‘negative’ if the cut-off point was less than the median score.

### Data processing and analysis

Data were cleaned and entered using Epidata version 4.6 and analyzed using SPSS version 26. Logistic regression was carried out to identify significant associated factors with OSCE implementation. Variables with p < 0.25 in the bivariate analysis were selected as candidate variables for the multivariate logistic analysis to control the effect of confounders. Adjusted odds ratios (AOR) with their 95% confidence interval and p < 0.05 were considered to have a significant association between the outcome and the independent variables. Model fitness was checked using Hosmer and Lemeshow, and it was found fit.

For the qualitative portion of the study, the thematic analysis method, as described by Braun and Clark [25], was employed for analysis and writing. This involved familiarizing with the data through repeated reading of the transcripts, generating initial codes, searching for potential themes and sub-themes, reviewing and refining themes and sub-themes, defining and naming themes and sub-themes, and producing a report based on their relevance and data capture.

Initially, the audio-recorded interviews were transcribed verbatim. Transcribed interviews were compared with field notes and proofread while listening to the audio recordings. The transcribed interview was translated into the English language by linguists, saved as a Word document, then converted to plain text and exported to Open Code software version 4.02 for coding and to perform further analysis. The transcriptions were read and reread to get an understanding of the data and obtain codes for thematic analysis. The data were organized in a meaningful way and coded to reduce the data volume.

### Ethics approval

This study has been approved by the Jimma University Institutional Review Board (Ref. number JUIH/IRB-345/2023) on 20 December 2023. Jimma University Institute of Health wrote a letter of permission to each university, and before starting actual data collection, permission was obtained from all universities. An information sheet was prepared and explained to study participants, including the purpose and importance of the study, the confidentiality of the participant’s response, and the benefits and risks of the study. Informed, voluntary, written, and signed consent was sought from the participants. The confidentiality of the data was guaranteed by using identification numbers rather than names and limiting access to the data.

## Results

### Sociodemographic characteristics of the respondents

Two hundred and sixty-nine educators were sampled for this study. Of the total sample size, 260 (97%) responded to the study. The median age and interquartile range (IQR) of the study participants were 29 ± IQR 6 years old. Among the respondents, 180 (69.2%) were male, and 152 (58.2%) were married. In terms of academic rank, 155 (60%) were lecturers, 150(57.6%) were from the nursing department, 140(53.9%) had less than 5 years, only 62 (23.8%) had trained in OSCE and 164 (63%) had previous experience in OSCE preparation. (Table 1)

**Table 1:** Sociodemographic characteristics of educators who participated in the study and worked in universities of the south and central region, Ethiopia, 2023(N = 260).

Variables	Category	Frequency	Percentage
Age in years	<25	23	8.8
	25-30	148	56.9
	31-35	54	20.8
	>35	35	13.5
Sex	Female	80	30.8
	Male	180	69.2
Religion	Protestant	118	45.4
	Orthodox	80	30.8
	Muslim	22	8.6
	Catholic	13	5.0
	*Other	28	10.8
Marital status	Single	100	38.5
	Married	152	58.5
	Divorced	8	3.1
Department	Midwifery	110	42.4
	Nursing	150	57.6
Job title/ academic rank	GA II	8	3.1
	Assistant lecturer	41	15.8
	Lecturer	156	60
	Assistant professor and above	55	21.2
Work experience in the year	1-5	140	53.9
	6-10	90	34.6
	Above 10	30	11.5
Training on OSCE	Yes	62	23.8
	No	198	76.2
Previous experience in OSCE preparation	Yes	164	63
	No	96	37

*Other=Apostolic, Jehovah Witness, GA II= Graduate Assistant II

OSCE Facility-Related Factors in the Universities of the South and Central Region

Regarding the use of OSCE as a formative assessment method, of the total of respondents, 122 (46.9%) used OSCE as a formative assessment method for all core competency courses and 59 (42.7%) did not, due to a lack of resources, staff shortage, and time constraints.

Ninety (34.6%) of the educators in total had OSCE implementation checklists available in their departments. Of the total of educators, 94 (36%) responded that the OSCE hall could occupy at least five stations; only 41 (15.8%) had the necessary equipment available at the station; and 102 (39.2%) responded that the department had a doll to facilitate the exam process. (Table 2)

**Table 2:** OSCE facility-related factors among educators who participated in the study and worked in the Universities of the South and Central Region, Ethiopia, 2023 (N = 260).

Variables	Category	Frequency	Percentage
Checklist about OSCE at the department or skill lab	Yes	90	34.6
	No	170	65.4
OSCE is a continuous assessment method for core competency courses.	Yes	122	46.9
	No	138	53.1
#If no, what is the reason?	lack of resources	59	42.7
	Lack of training	40	28.9
	*Other	39	28.4
If OSCE was used for continuous assessment, timely feedback offered to students	Yes	69	56.6
	No	53	43.4
Having the OSCE exam hall, which occupies at least 5 station	Yes	94	36
	No	166	64
Availability necessary equipment	Yes	41	15.8
	No	219	84.2
Standardized patient or doll to simulate the Case scenario	Yes	102	39.2
	No	158	60.8

OSCE = Objective Structured Clinical Examination, #multiple responses are possible, *Other = time consuming, staff shortage,

### Perceptions towards OSCE among participants

Only 122 (46.1%) educators had a positive perception of OSCE.

### Implementation of OSCE by Respondents

Regarding the implementation of OSCE among midwifery and nursing educators, 43.5% (95%CI: 37.4, 49.5) had a good implementation.

Educators agreed that all learning domains were covered by the checklist and that each item only represented one topic. 147 (56.5%) agreed and strongly agreed that the checklist items were appropriate and in line with the time allotted and the specified learning objectives. However, only a considerable number of them, 86 (33.1%), expressed their experience that they had used a predetermined scoring checklist, and 161 (61.9%) educators agreed that timely individualized feedback was offered on test performance. Of the educators, only 97 (37.3%) agreed that 5–10 stations were used; only 117 (45%) agreed that the names and objectives of the stations were clearly described and in line with the course objectives. Concerning time allocation, only 92 (35.4%) of the teachers agreed that 5–10 minutes were allowed per station.

### OSCE Implementation across Universities and Demographic characteristics

The implementation of OSCE was analyzed against various institutional and demographic characteristics of the faculty, revealing variations across Midwifery and Nursing departments and universities.

Faculty members in the Midwifery department demonstrated a higher rate of good OSCE implementation (50.9%, *n* = 58) compared to those in the Nursing department, where less than half (37.7%, *n* = 55) showed good implementation, Similarly, OSCE implementation varied significantly across the studied institutions. Wolkite University had the highest proportion of poor implementation (71.4%, *n* = 50). Conversely, Wachemo University and Arbaminch University achieved the highest rates of good implementation at 54.1% (*n* = 33) and 50.7% (*n* = 35), respectively, while Wolaita Sodo University stood at 41.7% (*n* = 25).

At Faculty member experience and academic Rank, No statistically significant differences in OSCE implementation were found based on the faculty members’ years of experience or academic rank. However, descriptive trends indicated that: Nearly half of faculty members with 1–5 years of experience (47.6%, *n* = 65) and 6–10 years of experience (41.4%, *n* = 36) demonstrated good OSCE implementation, whereas only 30.0% (*n* = 9) of those with more than 10 years of experience did so.

More than half of the junior staff, including Graduate Assistants II (62.5%, *n* = 5) and Assistant Lecturers (51.2%, *n* = 21), demonstrated good implementation. In contrast, less than half of Lecturers (41.9%, *n* = 65) and Assistant Professors and above (39.3%, *n* = 22) achieved good OSCE implementation (Table 3).

**Table 3:** OSCE Implementation across Universities and Demographic characteristics of educators who participated in the study and worked in universities of the south and central region, Ethiopia, 2023(N = 260).

Variables	Category	Implementation of OSCE		P–value
		Poor	Good	
Department	Midwifery	56(49.1)	58(50.9)	0.033
	Nursing	91(62.3)	55(37.7)	
Universities	Arbaminch University	34(49.3)	35(50.7)	0.014
	Wolaita Sodo	35(58.3)	25(41.7)	
	Wachemo	28(45.9)	33(54.1)	
	Wolkite	50(71.4)	20(28.6)	
Work experience	1-5 Years	75(52.4)	68(47.6)	0.188
	6-10 Years	51(58.6)	36(41.4)	
	>10 years	21(70)	9(30)	
Academic rank	Graduate assistant II	3(37.5)	5(62.5)	0.435
	Assistant lecturer	20(48.8)	21(51.2)	
	Lecturer	90(58.1)	65(41.9)	
	Assistant professor and above	34(60.7)	22(39.3)	

### Result of the qualitative part

In the qualitative part, 15 participants, 7 educators, and 8 students participated. The educators were in the age range of 29–36 years and were lecturers and above, and students were in the 19–27 years. Perceptions towards OSCE and its implementation, poor organization and planning, shortage of resources, and insufficient number and duration of stations were generated as themes as presented below.

Participants asked about their feeling about the inclusion of written stations along with the procedural ones, the majority of the students and examiners were against the idea despite the fact that students complained about a lack of awareness about OSCE principles. Regarding the overall process of the OSCE implementation, the majority of the participants leveled it as poor. Across the themes, ‘age of the student with grade level’ and ‘age of educators with academic rank’ stand for student and educator participants, respectively. The themes, expected to encompass the factors under investigation, were termed in a self-explanatory way for ease of understanding as follows.

**Perceptions towards OSCE and its implementation**: Based on the responses they provided during in-depth interviews, participants perceptions of the OSCE format’s structure vary from “very nice” to “not well-structured.” When describing their impression of the OSCE, participants frequently use expressions like happy with it, I like it, excited about it, assesses well, best, good, etc. Most of the respondents indicated that they perceived that OSCE was good.

*‘I like OSCE because it correlates with the main area of our field, of course.’* ***[4***^***th***^ ***year, 22 years old].*** *“It is the best strategy for developing and evaluating technical skills; it should be strengthened.”* ***[3***^***rd***^ ***year, 19 years old****]. “It is good and increases our confidence in performing procedures on patients.”* ***[4***^***th***^ ***year 21 years old].*** *Furthermore, OSCE was perceived as a method that comprehensively assesses clinical competence. For example, in this regard, an educator said: “... I think it is well organized and assesses them [their competence] very well.”* ***[27 years old lecturer].***

All educators were satisfied with the exam format, its significant effect on teaching and learning, and the way it was designed. However, students’ perceptions of the OSCE were somewhat affected by inadequate preparation time and resources, examiner interruptions, poor coordination, and a lack of feedback.

*‘[It is good; it has a good structure but lacks full coordination... time should be given adequately].’* ***[3rd-year student, 20 years old****]. ‘Inadequate preparation for the examination and the involvement of examiners during the exam contributes to stress’ (4th-year* ***student, 27 years old)***.

The implementation of OSCE status among participants varies according to their responses from ‘good’ to ‘not well structured or standardized and low quality.

*‘To tell the truth, the implementation of OSCE becomes problematic once you have a large class [large number of students]. I didn’t clearly understand how to use it as a teaching method, and it became difficult to get the appropriate ratio of teacher to student to give them timely feedback.’ (* ***31-year lecturer*** *).*

*‘We apply it [OSCE], even if it is not standardized. Because a lack of resources, training, time constraints, and a large number of students per class can affect the implementation. Station preparation, time allocation, feedback, orientation provision, and scoring were varied accordingly ‘[* ***32 years old, lecturer]’***

*Additionally, the students felt that the oral narration of the case scenarios caused bias and confusion before the task, and they perceived that the written case scenarios provided greater clarity than the oral narration.*

Educators in in-depth interviews expressed dissatisfaction with the difficulties they faced implementing the OSCE as a result of a lack of standardized rules and suggested that national preparation be done beforehand.

*“If a student studied or evaluated a method with 20 items, [the student] may not pass when he tested the same procedure with 30 items. This does not suggest the students’ potential. ‘[29-year-old lecturer].’ ‘Yes, it has a great influence [not having a standardized checklist] on the application of OSCE. If we have a nationally common standardized checklist, it is easy to apply. ‘[* ***30-year-old lecturer*** *].*

**Poor organization and planning:** Poor organization implies problems related to OSCE processes such as design, setting up, preparations, and running of OSCE. Overall, the organization of the OSCE was rated as good by both students and educators. However, the students responded that the preparation and organization of OSCE needed to be enhanced. In addition to not allocating enough time for each task, some problems with OSCE organization include unclear instructions and distracting or “shouting” examiners*.*

*Additionally, the students felt that the oral narration of the case scenarios caused bias and confusion before the task, and they perceived that the written case scenarios provided greater clarity than the oral narration.*

*‘While overall it was good, some of the instructions were not clear, and it would be better to make them clear. Some of the examiners were shouting.’* ***[3rd-year student, 23 years old].***

Moreover, students complained during in-depth interviews that they were not satisfied with the orientation provided, and they suggested that to familiarize themselves with the method of assessment, there had to be an orientation and practice OSCE session before the real exam.

*‘Most of us do not know how and what to prepare. We don’t know the resources to read about preparation [for OSCE]’ [4th-year* ***student, 25 years old]****. ‘It is better if we practice it before.’ [****4***^***th***^ ***year student, 22 years old****].*

**Shortage of resources:** Almost all KI, educators, and students complained that the absence of necessary and functional equipment in the lab could affect the implementation of OSCE. It limited the educator’s ability to select a representative sample; practicing with assumptions cannot help students develop clinical skills.

*“The shortage of instruments, or even if they were present but not functional, this differs from a real patient scenario.’* ***(34-year-old assistant professor).***

*‘Lack of resources, time constraints, a large number of students per class, and the majority did not train, lack of training affects its implementation [OSCE] in general.’ [32-year-old* ***lecturer****].* Poor planning and preparation for OSCE were perceived mainly as related to logistics and capacity building for examiners.

*‘I think it is better if the examiners are trained before the exam. A limited number of manpower [trained] and instruments [simulators and other lab equipment] are challenging. ‘[29 year’s* ***old, lecturer].***

*“The exam was overall fair, but despite the best efforts of departments and some teachers to standardize the exam, there was some variability between stations and between examiners, for example, in time allocation, scoring system, practice time and materials, and some teachers even did not respect the students when they made a small mistake. Practicing with assuming and communicating with dolls is very challenging and cannot help to develop clinical skills” [4th-year* ***student, 21 years old****].*

Also, the lack of an exam hall was mentioned as a main problem in selecting relevant cases with sufficient time allocation and providing appropriate individualized feedback to the students.

*“For four departments under the school of us [nursing school], we have only one skill lab, Also, it is not conducive to selecting enough cases with sufficient time allocation to provide appropriate individualized feedback. In addition, [the absence of an exam room] limits the ability to accurately assess students’ performance and provide a fair evaluation. Most of the time, students are evaluated at a single station.’ [33-years* ***old, lecturer****].*

*‘Examining stations 5 and above by providing appropriate time and feedback while having a large number of students in a narrow exam room is challenging. It also hinders the rotation from station to station with limited time, making it harder for students to fully engage with and demonstrate their knowledge in each area.’ [* ***36-year-old assistant professor*** *].*

**Insufficient number and duration of stations:** The number of stations in the sampled competencies was criticized by both educators and students. Maintaining a fair representation of competencies at OSCE stations was found to be a challenge for faculty.

One of the educators mentioned that it would be difficult even to imagine a representation of obstetric competence for 4th-year students only with two or three procedures such as normal labor management, AMTSL, and neonatal resuscitation.

*‘The topics covered are very important in obstetrics, and they are very common procedures…but [only] a limited number of stations were sampled.’ [4th* ***year student, 25 years old]***

Almost all of the educators agreed that the number of sampled competencies or skills was important, but there were too few to assess students against what they learned. The reasons for planning a lesser number of skills were the shortage of examiners and the lack of all necessary models and materials in the simulation center. Additionally, participants reported inadequate time to complete the stations. The students complained that there was not enough time allocated to each station to complete the given task completely. In this regard, some examiners concurred as well. They felt that even with fewer stations, there wasn’t enough time allotted for each station. However, the majority of the findings in this theme were from students and most of them reported that the time spent on each station was not sufficient to perform the given task completely

*“Time was not enough for the stations. I think it does not give us time to think and gather our thoughts. It’s not fair. Even we did not have time to think.’* ***[3rd year, 23 years old]*** *‘It is better to have enough stations with enough time.’* ***[4***^***th***^ ***year, 21 years].***

Overall, regarding the number and duration of the stations, responses show that study participants from the students’ group emphasized the shortage of time allocated for stations whereas educators perceived the unrepresentativeness of sampled stations as a challenge.

### Factors associated with OSCE implementation among respondents

In the multivariate logistic regression analysis, training on OSCE, availability of equipment, OSCE implementation checklist, and teacher perceptions were found to be significantly associated with OSCE implementation. Educators who had received OSCE training were twice as likely to implement OSCE compared to those who did not receive training (AOR = 2.07, 95%CI: 1.03, 4.171).

The odds of OSCE implementation among educators who have the necessary equipment in their skill laboratory were 2.36 times higher than those who do not have the necessary equipment (AOR = 2.36, 95% CI 1.03, 5.39). Similarly, educators who had an OSCE implementation checklist in their department were two times more likely than their counterparts (AOR = 1.89, 95% CI: 1.05, 3.42).

Having a positive perception of OSCE had a statistically significant relationship with the implementation of OSCE, and those who had negative perceptions about OSCE were 46.8% less likely to implement OSCE than those who had positive perceptions of OSCE. (AOR = 0.53, 95% CI 0.31, 0.92) (Table 4)

**Table 4:** Bivariate and multivariate logistic regression analysis of factors associated with OSCE implementation among midwifery and nursing educators in universities of the south and central region, Ethiopia, 2023(N = 260).

Variables		Implementation of OSCE		COR (95%CI)	AOR (95% CI)	P value
		Good	Poor			
Gender	Female	39(15.0)	41(15.8)	1.36(1.00, 2.31)*	1.21(0.66, 2.20)	P = 0.542
	Male	74(28.4)	106(40.8)	1	1	
Work Experience	1-5	68(26.1)	72(27.7)	2.20(1.09, 5.15)*	1.82(0.69, 4.78)	P = 0.225
	6-10	36(13.8)	54(20.8)	1.56(0.64, 3.78)	1.216(0.47, 3.17)	P = 0.690
	>10	9(3.5)	21(8.0)	1	1	
OSCE preparation experience	Yes	86(33.0)	78(30.0)	2.67(1.09, 5.55)*	2.77(0.93,7.47)	P = 0.054
	No	28(10.8)	68(26.2)	1	1	
Training OSCE	Yes	34(13.0)	28(10.8)	1.83(1.03, 3.25)	2.07(1.03, 4.17)	P = 0.042**
	No	79(30.4)	119(45.8)	1	1	
Availability of checklist	Yes	55(21.1)	35(13.5)	3.03(1.79, 5.15)	1.89(1.05, 3.42)	P = 0.037**
	No	58(22.3)	112(43.1)	1	1	
having exam hall	Yes	54(20.9)	40(15.1)	2.49(1.48, 4.19)*	1.67(0.92, 3.52)	P = 0.059
	No	59(22.9)	107(41.1)	1	1	
Availability of Equipment	Yes	28(10.8)	13(5.0)	3.39(1.67, 6.92)	2.36(1.03, 5.39)	P = 0.041**
	No	85(32.7)	134(51.5)	1	1	
Perceptions	Negative	50(19.5)	88(34.4)	0.53(0.32, 0.87)	0.53(0.31, 0.92)	P = 0.024**
	Positive	63(23.8)	59(22.3)	1	1	

Note: * Statistically significant at P-value<0.25, ** at p < 0.05, 95%CI, 1 = Reference group, OSCE = Objective Structured Clinical Examination, AOR = adjusted odds ratio, COR = Crude odds ratio.

Abbreviation: AOR: Adjusted odds ratio; CI, Confidence interval; COR, Crude odds ratio; IDI, In-depth interview; JU, Jimma University; KI, Key informant; OSCE, Objective structured clinical examinations; OSPE, Objective structured practical examination; SP, Standardized patients

During an in-depth interview, the investigator tried to address possible barriers at each respective university. Most key informants claim that there is no single responsible body for poor OSCE implementation. It requires the collaboration of all the entities involved at universities.

Every stakeholder has a significant and irreplaceable role in teaching and assessing the clinical performance of students. Major stakeholders raised by the discussants are the government, educators, technical assistants, and university management bodies, including the concerned nongovernmental organizations. Without coordinating the efforts of all these stakeholders, it is still difficult to perform in the expected way.

They also raised issues related to poor planning and coordination regarding schedules for practice and OSCE exams, a lack of standardization (nationally harmonized guideline), large numbers of students per class, and the negligence of educators to update existing checklists or develop new ones at the departmental level.

Another idea proclaimed by almost half of the participants focuses on the fact that poor orientation of students can stem from a lack of experience and training (a shortage of trained human power and a lower quality of training), a shortage of resources such as equipment, problems in the exam hall which leads to selecting insufficient number station, and the problem of time constraints.

## Discussion

This study revealed that only 43.5% (95% CI: 37.4, 49.5) of educators had a good OSCE implementation. Training about OSCE, having an OSCE checklist, the availability of necessary equipment, and positive perceptions towards OSCE were significantly associated factors with OSCE implementation. Perceptions towards OSCE and its implementation, poor organization and planning, shortage of resources, and insufficient number and duration of stations were the major findings.

According to this study, only 43.5% (95% CI 37.4, 49.5) of the educators had a good OSCE implementation. The results of a qualitative study support this finding. The study’s main promising findings were the positive perceptions of educators and students of the OSCE format. Student and examiner satisfaction with OSCE, the main reason for their preference was that OSCE truly evaluates their clinical skills and has more validity and reliability.

Despite the positive perceptions towards OSCE formats that encourage the implementation of OSCE in the future, the implementation of OSCE lacks several crucial elements such as adequate instruction and feedback, sufficient number and duration of stations, proper planning and preparation for OSCE, and the appropriate scoring that this study revealed.

This study suggests that the OSCE implementation process lacks proper organization and planning. Poor organization is a finding related to the failure to plan adequately for the OSCE exam. During the planning phase, the OSCE team should schedule meetings and training sessions before the exam day to prepare the exam blueprint, design stations, develop scenarios, orient examiners, set up rooms and equipment, and handle other management concerns.

Educators and students both raised complaints about issues that were either directly or indirectly attributed to poor organization. Interfering educators, uncertain instructions, inadequate station and time allocation, and lack of materials were among the issues mentioned in the reports. Khan et al. proposed sharing responsibility among the team, exam schedule preparation, rules and regulations, preparation of the exam blueprint, determination of the station number and duration of the exam, development of the intended OSCE stations, preparation of the marking tool, and, lastly, piloting of the circuits developed before the OSCE exam to handle poor organization and related issues [24].

Generally, the findings show that the implementation of OSCE is not being applied properly. This limits the interaction between patients and students during their clinical integration and their acquisition of the competencies required by the competency-based curriculum implementation approach.

This suggests that more than half of educators do not implement OSCE according to the OSCE quality assurance criteria in the exam implementation guide of the Minister of Health of Ethiopia [7], this might be due to the short duration of time since its (OSCE) implementation started, and it needs time to scale up, create positive perceptions among educators and collaboration from all concerned bodies.

In addition, the narrow time gap between this study and the amendment of the exam manual could be a significant reason why the introduction of OSCE implementation at the Ethiopian Public University has recently started.

The findings of this study revealed that OSCE training was significantly associated with its implementation. Educators who had been trained were twice as likely to implement OSCE than those who had not been trained.

This finding is supported by the findings of the qualitative study. During in-depth interviews, from the examiner’s side, the challenge of planning and preparation for OSCE was mainly perceived as related to logistics and the capacity building of the examiners. Training is essential to run OSCE, however, educators complained that the majority of the staff received any kind of training on OSCE, which can contribute to lowering its OSCE quality.

This finding was well in agreement with previous studies conducted in Addis Ababa, St. Paul’s Millennium Medical College, and the University of Caliber, Nigeria [18,22]. This could be because attending training can help educators gain more understanding of how to prepare the case scenario and run OSCE during the exam, allowing them to better implement. It can also lead to better standardization of the exam scoring This highlights the need for examiner training [26].

The result of this study revealed statistical significance between the implementation of OSCE by educators and the existence of a checklist; those who have a checklist implemented are two times more likely than their counterparts. Educators at IDI complained that they encountered problems in implementing OSCE due to a lack of harmonized standard guidelines and recommended that they be prepared at the national level. It makes it easy to implement OSCE and helps to assess students objectively.

This is consistent with the qualitative studies conducted at Jimma, the Caliber University of Nigeria, and Addis Ababa Paul’s Medical College [17,18,22]. It might be that the guideline guides educators in how, when, and which case scenarios should be selected for the exam; it also helps educators build confidence and increase their readiness to implement OSCE.

Another factor statistically related to the implementation of OSCE by educators was the availability of the necessary equipment. Those who have the necessary equipment in the skill lab were 2.3 times more likely to implement it compared to those who do not have equipment in the skill lab. Qualitative results complement this as well.

During the in-depth interview, almost all educators and students complained that the absence of necessary and functional equipment at the lab could affect the OSCE implementation. It limited the educator’s ability to select a representative sample; practicing with assumptions cannot help students develop clinical skills.

Most educators and students revealed that there was a shortage of instruments, or even if they were present, they were not functional; this differs from a real patient scenario and affects the clinical skill development of the students. It reduces the quality of OSCE implementation.

An even higher percentage reported it as a challenge previously in studies done in India, Riyadh University of Saudi Arabia, and Egypt [9,27,28], This might be because OSCE is a very demanding examination method, requiring an abundant amount of human and material resources [22].

Resource limitations in healthcare training often arise from inadequate policy frameworks or misaligned funding allocations, which fail to address the growing demands of medical education. These structural deficiencies are further compounded by sociocultural norms that shape perceptions of medical careers, sometimes discouraging potential candidates due to perceived prestige, financial instability, or excessive workload. In low- and middle-income countries (LMICs), these challenges are intensified by systemic weaknesses, including fragmented faculty development programs and unequal distribution of training resources.

Without robust policy interventions, these disparities persist, leading to uneven access to quality medical education. The resulting shortage of skilled healthcare professionals not only strains health systems but also diminishes patient outcomes, as understaffed facilities struggle to meet care standards. Addressing these issues necessitates a holistic strategy that integrates policy reform, sustainable financing, and shifts in societal attitudes toward medical professions.

Universities must make the availability of simulation materials a priority. A well-organized and complete simulation lab should be prepared both for practice and for final assessment.

Similarly, respondents’ perceptions were significantly associated with their implementation of OSCE; those educators with a negative perception towards the OSCE, implementing OSCE were 46.8% less likely compared with those with a positive perception.

This is supported by studies from Pakistan, which report that those who have positive perceptions towards OSCE recommend that it be used in the future and would like it to apply to all batches, both by students and examiners. This may be because educators will feel more motivated to apply the OSCE if they have positive perceptions [24].

A sustainable solution to these barriers requires coordinated efforts among governments, educational institutions, and healthcare providers to implement equitable and long-term reforms. Policymakers must prioritize faculty development through structured training initiatives, while funding mechanisms should ensure fair resource distribution across all training centers. Additionally, partnerships between academic institutions and healthcare systems can help align curricula with workforce needs, ensuring graduates are well-prepared for real-world challenges. Beyond structural changes, shifting sociocultural narratives around medical careers through mentorship programs, educator awareness creation, and incentives can help attract and retain talent.

Without such multifaceted interventions, disparities in medical training will continue to undermine healthcare delivery, particularly in LMICs where systemic gaps are most acute. By embedding these strategies into national health and education policies, stakeholders can foster a more resilient and inclusive healthcare workforce, ultimately improving both training quality and patient care.

The findings reveal variations in OSCE implementation across departments and universities, with Midwifery faculty achieving higher implementation rate than Nursing, this may be due to midwifery’s more procedural, directly observable competencies, which afford greater exposure to skills-lab-based, structured assessment [17]; the marked variation across universities, with Wolkite performing poorer and Wachemo and Arbaminch good, may reflects less number examiner training sessions, shortage of resources including human power, and organizational readiness than any single faculty characteristic [29,30]. While teaching experience and academic rank showed no statistically significant differences, descriptive trends reveal that junior faculty and those with under five years of experience exhibited higher implementation quality than senior academics. The difference may be Newer faculty may benefit from recent training in competency-based education and are often directly tasked with running exam stations, keeping them familiar with standardized checklists. In contrast, senior faculty often face competing administrative or research obligations that limit their participation in calibration workshops, leading to a greater reliance on /adaptation with/traditional assessment methods.

### Implications of the Study

In general, the results of the current study indicate that less than half of teachers have a good OSCE implementation status, highlighting the need for improved training, support, and resource allocation for effective medical education. Ethiopian public universities in the central and southern regions should address gaps in OSCE implementation by allocating resources and providing effective training for educators. This will improve the quality of healthcare education and ensure fair assessments, leading to better learning outcomes. The study findings can inform policy design, standardized guidelines, clear expectations, and regular evaluations.

### Strengths and limitations of the study

The study was used at the regional level and incorporated mixed methods of data collection, including qualitative data from educators and students, to understand OSCE implementations and challenges, enhancing the credibility of the findings. However, the study did not use observational methods to observe educators’ implementation of OSCE, potentially leading to social desirability bias in self-reported data. It is a key limitation of this study, in which participants may over-report favorable attitudes or behaviors as a result of perceived social expectations. Regional focus may limit generalizability, cross-sectional design prevents causal inference, and we suggest using validated scales to reduce bias, triangulating with observational methods, or including anonymous response options in future research to address it.

## Conclusion

The study found that less than half of the south and central Ethiopian university teachers had a good OSCE implementation, with factors such as training, equipment availability, and educators’ perceptions affecting their implementation. Issues such as poor organization, planning, resource scarcity, and an insufficient number and duration of stations were identified as challenges in the OSCE implementation through a qualitative study.

To overcome the identified challenges, We recommend, using national standardization of OSCE checklists to ensure consistency in assessment; Implementation of faculty development programs, such as workshops on simulation-based training, providing OSCE training to the educators, and University stakeholders should ensure the availability of simulation infrastructure, other necessary equipment’s, adequate OSCE hall to address resource constraints. Also the University should implement mandatory OSCE calibration workshops and establish cross-departmental standardization modeled with high performing programs.

Midwifery and nursing educators must be familiar with institutional policies and implement OSCE with proper planning and organization. Validity should be ensured by focusing on the curriculum, important procedures, and clear instructions. Staying up-to-date on research and best practices ensures effective evaluation methods. Future research should employ observational methodologies to evaluate the implementation status and challenges experienced by educators. Furthermore, given that the current study was not designed to examine inter-departmental, institutional, or subgroup variations, multi-institutional comparative studies are recommended to directly observe examiner behavior, pinpoint operational barriers, and establish robust national assessment benchmarks.

## Abbreviation:

AOR: Adjusted odds ratio
CI: Confidence interval
COR: Crude odds ratio
IDI: In-depth interview
JU: Jimma University
KI: Key informan
OSCE: Objective structured clinical examinations
OSPE: Objective structured practical examination
SP: Standardized patients
