# Supplementary material for: Implementation of objective structured clinical examination among midwifery and nursing educators at South and Central Region Universities in Ethiopia, 2023
Source: PLoS One. 2026 Aug 26;21(8):e0356551. doi: 10.1371/journal.pone.0356551 (PMC13514063; doi:10.1371/journal.pone.0356551)
Supplement: Supplementary file 1 [file pone.0356551.s001.pdf]

PLOS ONE

Implementation of Objective Structured Clinical Examination and Associated Factors among Midwifery and Nursing Educators at South and Central Region Universities in Ethiopia, 2023

--Manuscript Draft--

Manuscript Number:	PONE-D-24-18847
Article Type:	Research Article
Full Title:	Implementation of Objective Structured Clinical Examination and Associated Factors among Midwifery and Nursing Educators at South and Central Region Universities in Ethiopia, 2023
Short Title:	Objective Structured Clinical Examination implementation and associated factors
Corresponding Author:	Zerihun Demessie, MSc<br>Hossana College of Health Sciences<br>Hossana, ETHIOPIA
Keywords:	implementation; Objective structured clinical examination SCE; education; Ethiopia
Abstract:	Introduction<br><br>The Objective Structured Clinical Examination (OSCE) is a well-known clinical skill evaluation tool that has been applied worldwide to teach and assess learners' competencies in healthcare disciplines. Poor implementation of OSCE, can lead to inaccurate assessments and ultimately result in the production of incompetent professionals. However, there is no evidence of the implementation status and factors that limit the much-needed OSCE practice in universities.<br><br>Method<br><br>An institution-based cross-sectional study with a mixed approach was used among 269 midwifery and nursing educators and 15 key informants from 8 May to 10 June 2023. All midwifery and nursing educators were included from randomly selected universities in southern and central Ethiopia. Quantitative data was collected using pretested self-administered questionnaires. To find independent factors, logistic regression analysis was used. With a p-value of less than 0.05, which denotes statistical significance, a corresponding 95% confidence interval (CI) was calculated. Qualitative data was collected through in-depth interviews and analyzed using open-code software Version 4.02, and findings were used to support quantitative findings.<br><br>Result<br><br>A total of 260 (97%) educators participated in this study, and 43.5% (95% CI 37.4-49.5) of them had a good OSCE implementation. Training in OSCE (AOR= 2.07, 95% CI 1.029-4.17), having an OSCE checklist (AOR= 1.893, 95% CI 1.052-3.420), having equipment (AOR = 2.360, 95% CI 1.032-5.395) and negative perceptions toward OSCE (AOR= 0.532, 95% CI 0.307-0.921) were significantly associated, and poor organization and planning, perceptions towards OSCE, shortage of resources and insufficient number and duration of the station were found by qualitative study.<br><br>Conclusion<br><br>Less than half of educators have had a good OSCE implementation. Training, equipment, checklists, and perceptions highlight issues such as poor organization and planning, resource, and time allocation stations found as influencing factors. In addition to improving in-service and ongoing OSCE training, it is preferable to ensure the availability and accessibility of resources in the facilities to improve OSCE implementation.
Order of Authors:	Zerihun Demessie
	Bekena Fekecha, PhD

Powered by Editorial Manager® and ProduXion Manager® from Aries Systems Corporation

	Kebenesa Angasu
**Additional Information:**	
**Question**	**Response**
**Financial Disclosure**<br><br>Enter a financial disclosure statement that describes the sources of funding for the work included in this submission. Review the submission guidelines for detailed requirements. View published research articles from *PLOS ONE* for specific examples.<br><br>This statement is required for submission and **will appear in the published article** if the submission is accepted. Please make sure it is accurate.<br><br>**Funded studies**<br>Enter a statement with the following details:<br>• Initials of the authors who received each award<br>• Grant numbers awarded to each author<br>• The full name of each funder<br>• URL of each funder website<br>• Did the sponsors or funders play any role in the study design, data collection and analysis, decision to publish, or preparation of the manuscript?<br><br>Did you receive funding for this work?	The author(s) received no specific funding for this work.
**Competing Interests**<br><br>Use the instructions below to enter a competing interest statement for this submission. On behalf of all authors, disclose any competing interests that could be perceived to bias this work—acknowledging all financial support and any other relevant financial or non-financial competing interests.<br><br>This statement is required for submission and **will appear in the published article** if the submission is accepted. Please make sure it is accurate and that any funding	the authors have declared that no competing interests exist.

sources listed in your Funding Information later in the submission form are also declared in your Financial Disclosure statement.<br><br>View published research articles from *PLOS ONE* for specific examples.<br><br>**NO authors have competing interests**<br><br>Enter: *The authors have declared that no competing interests exist.*<br><br>**Authors with competing interests**<br><br>Enter competing interest details beginning with this statement:<br><br>*I have read the journal's policy and the authors of this manuscript have the following competing interests: [insert competing interests here]*<br><br>* typeset	
**Ethics Statement**<br><br>Enter an ethics statement for this submission. This statement is required if the study involved:<br><br>• Human participants<br>• Human specimens or tissue<br>• Vertebrate animals or cephalopods<br>• Vertebrate embryos or tissues<br>• Field research<br><br>Write "N/A" if the submission does not require an ethics statement.<br><br>General guidance is provided below. Consult the submission guidelines for detailed instructions. **Make sure that all information entered here is included in the Methods section of the manuscript.**	no

**Format for specific study types**<br><br>**Human Subject Research (involving human participants and/or tissue)**<br>• Give the name of the institutional review board or ethics committee that approved the study<br>• Include the approval number and/or a statement indicating approval of this research<br>• Indicate the form of consent obtained (written/oral) or the reason that consent was not obtained (e.g. the data were analyzed anonymously)<br><br>**Animal Research (involving vertebrate animals, embryos or tissues)**<br>• Provide the name of the Institutional Animal Care and Use Committee (IACUC) or other relevant ethics board that reviewed the study protocol, and indicate whether they approved this research or granted a formal waiver of ethical approval<br>• Include an approval number if one was obtained<br>• If the study involved *non-human primates*, add *additional details* about animal welfare and steps taken to ameliorate suffering<br>• If anesthesia, euthanasia, or any kind of animal sacrifice is part of the study, include briefly which substances and/or methods were applied<br><br>**Field Research**<br><br>Include the following details if this study involves the collection of plant, animal, or other materials from a natural setting:<br>• Field permit number<br>• Name of the institution or relevant body that granted permission	
**Data Availability**<br><br>Authors are required to make all data underlying the findings described fully available, without restriction, and from the time of publication. PLOS allows rare exceptions to address legal and ethical concerns. See the PLOS Data Policy and FAQ for detailed information.	Yes - all data are fully available without restriction

<table>
<tr><td>A Data Availability Statement describing where the data can be found is required at submission. Your answers to this question constitute the Data Availability Statement and will be published in the article, if accepted.

Important: Stating ‘data available on request from the author’ is not sufficient. If your data are only available upon request, select ‘No’ for the first question and explain your exceptional situation in the text box.

Do the authors confirm that all data underlying the findings described in their manuscript are fully available without restriction?</td><td></td></tr>
<tr><td>Describe where the data may be found in full sentences. If you are copying our sample text, replace any instances of XXX with the appropriate details.

• If the data are held or will be held in a public repository, include URLs, accession numbers or DOIs. If this information will only be available after acceptance, indicate this by ticking the box below. For example: All XXX files are available from the XXX database (accession number(s) XXX, XXX.).
• If the data are all contained within the manuscript and/or Supporting Information files, enter the following: All relevant data are within the manuscript and its Supporting Information files.
• If neither of these applies but you are able to provide details of access elsewhere, with or without limitations, please do so. For example:

Data cannot be shared publicly because of [XXX]. Data are available from the XXX Institutional Data Access / Ethics Committee (contact via XXX) for researchers who meet the criteria for access to confidential data.

The data underlying the results presented in the study are available from (include the name of the third party</td><td>in the manuscript</td></tr>
</table>

and contact information or URL).<br>• This text is appropriate if the data are owned by a third party and authors do not have permission to share the data.<br><br>* typeset	
Additional data availability information:	Tick here if the URLs/accession numbers/DOIs will be available only after acceptance of the manuscript for publication so that we can ensure their inclusion before publication.

Powered by Editorial Manager® and ProduXion Manager® from Aries Systems Corporation

Manuscript

Click here to access/download;Manuscript;Manuscrip-(OSCE).docx

Implementation of Objective Structured Clinical Examination and Associated Factors among Midwifery and Nursing Educators at South and Central Region Universities in Ethiopia, 2023

Zerihun Demessie[1*], Dr. Bekena Fekecha[2&], Kebenesa Angasu[2&]

[1*] Department of Midwifery, Hossana Health Science College, Hossana, Ethiopia, [2&] Department of Midwifery, Jimma University, Jimma, Ethiopia.

*Correspondence Author:

Tel: +251927138769

Email: zerishdem3875@gmail.com

[&] These authors contributed equally to this work.

Abstract

Introduction: The Objective Structured Clinical Examination (OSCE) is a well-known clinical skill evaluation tool that has been applied worldwide to teach and assess learners' competencies in healthcare disciplines. Poor implementation of OSCE, can lead to inaccurate assessments and ultimately result in the production of incompetent professionals. However, there is no evidence of the implementation status and factors that limit the much-needed OSCE practice in universities.

Objective: To assess OSCE implementation and associated factors among midwifery and nursing educators in the South and Central region of Ethiopian universities, 2023.

Methods: An institution-based cross-sectional study with a mixed approach was used among 269 midwifery and nursing educators and 15 key informants from 8 May to 10 June 2023. All midwifery and nursing educators were included from randomly selected universities in southern and central Ethiopia. Quantitative data was collected using pretested self-administered questionnaires. To find independent factors, logistic regression analysis was used. With a p-value of less than 0.05, which denotes statistical significance, a corresponding 95% confidence interval (CI) was calculated. Qualitative data was collected through in-depth interviews and analyzed using open-code software Version 4.02, and findings were used to support quantitative findings.

Results: A total of 260 (97%) educators participated in this study, and 43.5% (95% CI 37.4-49.5) of them had a good OSCE implementation. Training in OSCE (AOR= 2.07, 95% CI 1.029-4.17), having an OSCE checklist (AOR= 1.893, 95% CI 1.052-3.420), having equipment (AOR = 2.360, 95% CI 1.032-5.395) and negative perceptions toward OSCE (AOR= 0.532, 95% CI 0.307-0.921) were significantly associated, and poor organization and planning, perceptions

towards OSCE, shortage of resources and insufficient number and duration of the station were found by qualitative study.

Conclusions: Less than half of educators have had a good OSCE implementation. Training, equipment, checklists, and perceptions highlight issues such as poor organization and planning, resource, and time allocation stations found as influencing factors. In addition to improving in-service and ongoing OSCE training, it is preferable to ensure the availability and accessibility of resources in the facilities to improve OSCE implementation.

Keywords: Implementation, OSCE, Education, Ethiopia.

Introduction

The Objective Structured Clinical Examination (OSCE) is a versatile multipurpose evaluative tool that can be utilized to assess the clinical competency of healthcare professionals, with significant consideration given to the examination's objectivity [1]. OSCE was first introduced in medical education in 1975 by Ronald Harden at the University of Dundee to improve the validity and reliability of performance assessment, as it is a widely used method for teaching and assessing student competencies worldwide, particularly in the healthcare field. It is a method for evaluating students' clinical competencies impartially and fairly [2–4].

OSCE is a multiple-station examination in which all learners must perform several clinical tasks within a specified time frame, and all exams are observed with standardized scoring rubrics in simulated environments [3,5]. It assesses learners' skills in history taking, physical examination, communication skills, patient management, diagnosis, equipment usage in experiments, sequence of steps followed in experiments, and data interpretation [6]. The stations are organized in such a way

that it allows students to rotate smoothly at a predetermined time while being observed by an examiner on a one-to-one basis using a simulator [7].

The OSCE assessment can be used for a formative assessment or a summative assessment. Both types of assessment are essential to promote learning and improve patient safety. Using the OSCE assessment, educators can encourage students to engage in active learning and critical thinking, which are crucial to developing safe and effective healthcare practices [8,9]. It is a gold standard, requiring a plan that translates learning objectives into clinical competencies such as history taking, physical examination, and technical skills. OSCE has a good balance of acceptability, reliability, validity, credibility, feasibility, cost, and educational impact. However, its use is expensive and time-consuming [10].

When OSCE is accompanied by appropriate feedback, it can be used as a learning purpose in addition to assessments [11,12]. In clinical skills practice, OSCE assessment had better learning outcomes compared to some other skill teaching methods, and the acquired skills last longer [11,13].

According to currently available evidence, OSCE is generally a more powerful, valid, highly reliable, and objective technique for evaluating students' skills than other conventional assessment formats like short- or long-case exams [13–15].

Effective management and understanding of potential issues make OSCE the most effective method for clinical assessment of competence. However, there have also been other difficulties with OSCE implementation. It appears to lack appropriate

staffing, and the few clinical instructors and tutors present probably lack the necessary competence to carry out OSCE [16].

The lack of standardization on the scoring sheet, the attitude and perception of students and examiners, and the lack of human and material resources all contribute to stress, student and examiner fatigue during the OSCE, a lack of time at the station and preparation before the exam, and others resulted in poor implementation of OSCE. [13,16–19] .

In Ethiopia, the OSCE has a relatively recent history. Institutions and stakeholders have worked intensively since the start to ensure OSCE implementation [20]. However, its development is not up to national expectations, even despite the recent government demand to include it in the format of a healthcare license exam and to employ it as a formative and summative assessment tool within the competency-based curriculum of the undergraduate midwifery and nursing program and other health disciplines [16,20,21].

Following each OSCE session held at a university, issues relating to the lack of theoretical and practical skills to conduct OSCE and the limits related to resources were often observed. Although some quantitative survey studies support the usage of OSCE in the country in terms of staff and student perspectives and attitudes [16,20,22], however, as far as my searching ability, no study has been conducted to assess the implementation status and perceived challenges and factors that affect the much needed OSCE in Ethiopian universities and colleges, particularly in the study area.

Therefore, this study aimed to answer the research question: what does the implementation of OSCE status look like? By exploring and assessing the gaps and challenges that affect OSCE implementation from the perspective of midwifery and nursing educators in the southern and central regions of Ethiopian universities. The findings help universities and other concerned bodies identify gaps and areas for improvement and develop strategies to better support the use or implementation of OSCEs as a teaching and learning evaluation tool in the future.

Methods

Setting and design of the study

This study was carried out in the South and Central regions of the Ethiopian University School of Midwifery and Nursing. Currently, there are six public universities in the south and central regions regulated by the Federal Ministry of Education. Arba Minch, Wolaita Sodo, Wachemo, Worabe, Dila, and Wolkite Universities. The distance from Addis Ababa to these institutions is approximately 430 km, 317 km, 230 km, 176 km, 351, and 166 km, respectively. The study was carried out between 8 May and 10 June 2023. An institution-based cross-sectional study was used that uses quantitative data supported by a qualitative study.

All midwifery and nursing educators, midwifery and nursing students who were third year or above from Ethiopian universities in the south and central regions were the source population. Participants who were recruited as regular academic staff from south and central Ethiopian universities with <6 months of work experience and who have a BSc degree and above were included.

The sample size and sampling methods were carefully designed to collect quantitative and qualitative data effectively. For the quantitative aspect, four universities (i.e., Wachemo, Wolkite, Arba Minch, and Wolaita Sodo universities) were randomly selected out of the six universities under study in the south and central regions. Interestingly, all 269 midwifery and nursing educators from these universities were included in the research sample. Given the relatively small number of study participants, opting for a census method was practical and ensured comprehensive coverage.

However, for qualitative data collection, a purpose-sampling method was used. This involved the deliberate selection of 15 key informants (KI) based on their years of experience and their positions within the school. Additionally, specifically, to gain a more nuanced perspective, class representatives and students in their third year or higher were selected for in-depth interviews. The determination of the qualitative sample size followed the principle of data saturation, where interviews were conducted until the responses became repetitive, ensuring that the collected data were sufficient to address the study objectives in full.

Data Collection and Procedure

The questionnaire was developed after reviewing different types of literature and making them fit the objectives of the study [7,16,17,20–22]. Tools were pre-tested among 26 educators and students who were not a part of the main study. The tools had five sections: sociodemographic, facility-related characteristics, perception toward OSCE, and OSCE

implementation. Two semi-structured in-depth interview guides were used (one for educators and the other for students), and they contained different questions.

For quantitative data collection, a pre-tested self-administered questionnaire was used. Data were collected by four data collectors (BSc Midwife) and four supervisors (Master of Science degree in public health), i.e., one data collector and one supervisor for each university. We chose them based on their experience with data collection. Then they were trained on the objectives of the study, the survey, and the data collection process. Principal investigators and supervisors double-checked the surveys to ensure that they were complete and relevant.

Qualitative data collection, In-depth interviews were conducted to collect data through face-to-face interactions. All interviews were conducted in separate locations where others were unable to listen to the interviews. The interviews lasted between 60 and 80 minutes. Data were recorded audio with the permission of study participants, and field notes (handwritten) were taken to take non-recordable gestures. Probes were used to obtain detailed information from the respondents whenever necessary. The interviews continued until the data saturation level.

Quality Control

Internal consistency (reliability) was tested by calculating the Cronbach Alpha using the SPSS version 26 window. Additionally, health behavior experts cross-checked the validity of the tool's content. Data collectors and supervisors were trained in the study instrument and data collection procedure. The principal investigator and the supervisors checked the completeness of the collected data.

In this study, the credibility of the qualitative study was ensured using the triangulation of data, transferability was ensured by a clear and thick description of the study, and the interview guide was developed without being influenced or dependent extensively on literature review evidence, dependability through conducting peer debriefings among data collectors to get their alternative explanations and similar consensus on emerging codes and to evaluate whether or not the findings are supported by data. Conformability was ensured through bracketing (that is, the interpretations of the data were derived from the collected data and not based on the research's viewpoints).

Measurement and Operational Definitions of the Variables

The implementation of OSCE was evaluated using the questionnaire developed from an OSCE implementation guide of the Ethiopian Ministry of Health. This questionnaire consisted of 11 items with 5-point Likert scale items. Educators were asked to rate on a five-point scale (1= strongly disagree, 2 = disagree, 3 = neutral, 4 = agree, 5 = strongly agree), how they implement the OSCE.

The overall OSCE implementation score (new variable) was calculated from the sum of the responses of the respondents for each item related to the implementation. The composite variable 'implementation status' was then dichotomized using the median score as the cutoff point. The median score was 33. Thus, respondents who scored the median score or higher were classified as having a good OSCE implementation and otherwise poor [7] .

Perception towards OSCE implementation has 11 items that were measured by using a Likert scale where educators were supposed to strongly agree, agree, neutral, disagree, and strongly disagree [23]. The overall perception score (new variable) was calculated from the sum

of the responses of the respondents for each item related to perception. Then the perceptions of the OSCE of the respondents were dichotomized using the median score as the cutoff point. The median score was 42. The perceptions of the respondents were considered 'positive' if the cut-off point was equal to the median score and above and 'negative' if the cut-off point was less than the median score.

Data Processing and Analysis

Data were cleaned and entered using Epidata version 4.6 and analyzed using SPSS version 26. Logistic regression was carried out to identify significant associated factors with OSCE implementation. Variables with $p < 0.25$ in the bivariate analysis were selected as candidate variables for the multivariate logistic analysis to control the effect of confounders. Adjusted odd ratios (AOR) with their 95% confidence interval and $p < 0.05$ were considered to have a significant association between the outcome and the independent variables. Model fitness was checked using Hosmer and Lemeshow and it was found fit.

For the qualitative portion of the study, the thematic analysis method, as described by Braun and Clark [24] , was employed for analysis and writing. This involved familiarizing with the data through repeated reading of the transcripts, generating initial codes, searching for potential themes and sub-themes, reviewing and refining themes and sub-themes, defining and naming themes and sub-themes, and producing a report based on their relevance and data capture.

Initially, the audio-recorded interviews were transcribed verbatim. Transcribed interviews were compared with field notes and proofread while listening to the audio recordings. The transcribed interview was translated into the English language by linguists, saved as a Word document, then converted to plain text and exported to Open

Code software version 4.02 for coding and to perform further analysis. The transcriptions were read and reread to get an understanding of the data and obtain codes for thematic analysis. The data were organized in a meaningful way and coded to reduce the data volume.

Results

Sociodemographic characteristics of the respondents

Two hundred and sixty-nine educators were sampled for this study. Of the total sample size, 260 (97%) responded to the study. The median age and interquartile range (IQR) of the study participants were 29 ± IQR 6 years old. Among the respondents, 180 (69.2%) were male, and 152 (58.2%) were married. In terms of academic rank, 155 (60%) were lecturers, 150(57.6%) were from the nursing department, 140(53.8%) had less than 5 years, only 62 (23.8%) had trained in OSCE and 164 (63%) had previous experience in OSCE preparation. **(Table 1)**

In the qualitative part, 15 participants, 7 educators, and 8 students participated. The teachers were in the age range of 29 to 36 years and were lecturers and above, and students were in the 19 to 27 years. The primary documents with 24 pages were analyzed, which were coded into 45 codes, and grouped into 4 themes and 12 subthemes. Perceptions towards OSCE and its implementation, poor organization and planning, shortage of resources, and insufficient number and duration of stations were generated as themes. **(Table 2)**

OSCE Facility-Related Factors in the Universities of the South and Central Region

Regarding the use of OSCE as a formative assessment method, of the total of respondents, 122 (46.9%) used OSCE as a formative assessment method for all core competency courses and 59 (42.7%) did not, due to a lack of resources, staff shortage, and time constraints.

According to the result generated from the in-depth interview, the difficulty of planning and preparation for OSCE was perceived mainly as related to logistics and capacity building for examiners.

'I think it is better if the examiners are trained before the exam. A limited number of manpower [trained] and instruments [simulators and other lab equipment] are challenging. '[29 year's ***old, lecturer].***

Ninety (34.6%) of the educators in total had OSCE implementation checklists available in their departments. Of the total of educators, 94 (36%) responded that the OSCE hall could occupy at least five stations; only 41 (15.8%) had the necessary equipment available at the station; and 102 (39.2%) responded that the department had a doll to facilitate the exam process. **(Table 3)**

Almost all KI, educators, and students complained that the absence of necessary and functional equipment in the lab could affect the implementation of OSCE. It limited the educator's ability to select a representative sample; practicing with assumptions cannot help students develop clinical skills.

"The shortage of instruments, or even if they were present but not functional, this differs from a real patient scenario'. ***(34-year-old assistant professor).***

'Lack of resources, time constraints, a large number of students per class, and the majority did not train, lack of training affects its implementation [OSCE] in general.' [32-year-old ***lecturer****].*

"The exam was overall fair, but despite the best efforts of departments and some teachers to standardize the exam, there was some variability between stations and between examiners, for example, in time allocation, scoring system, practice time and materials, and some teachers even did not respect the students when they made a small mistake. Practicing with assuming and communicating with dolls is very challenging and cannot help to develop clinical skills" [4th-year ***student, 21 years old****].*

Educators in in-depth interviews expressed dissatisfaction with the difficulties they faced implementing the OSCE as a result of a lack of standardized rules and suggested that national preparation be done beforehand.

*"If a student studied or evaluated a method with 20 items, [the student] may not pass when he tested the same procedure with 30 items. This does not suggest the students' potential. '[29-year-old lecturer].' 'Yes, it has a great influence [not having a standardized checklist] on the application of OSCE. If we have a nationally common standardized checklist, it is easy to apply. ' [****30-year-old lecturer****].*

Also, during the in-depth interview, the lack of an exam hall was mentioned as a main problem in selecting relevant cases with sufficient time allocation and providing appropriate individualized feedback to the students.

'For four departments under the school of us [nursing school], we have only one skill lab, Also, it is not conducive to selecting enough cases with sufficient time allocation to provide appropriate individualized feedback. In addition, [the absence of an exam room] limits the ability to accurately assess students' performance and provide a fair evaluation. Most of the time, students are evaluated at a single station. ' [33-years ***old, lecturer****].*

*'Examining stations 5 and above by providing appropriate time and feedback while having a large number of students in a narrow exam room is challenging. It also hinders the rotation from station to station with limited time, making it harder for students to fully engage with and demonstrate their knowledge in each area.' [****36-year-old assistant professor****].*

Perceptions towards OSCE among participants.

Only 122 (46.1%) educators had a positive perception of OSCE. Based on the responses they provided during in-depth interviews, participants' perceptions of the OSCE format's structure vary from "very nice" to "not well-structured". When describing their impression of the OSCE, participants frequently use expressions like happy with it, I like it, excited about it, assesses well, best, good, etc. Most of the respondents indicated that they perceived that OSCE was good.

'I like OSCE because it correlates with the main area of our field, of course.' ***[4th year, 22 years old].*** *"It is the best strategy for developing and evaluating technical skills; it should be strengthened."* ***[3rd year, 19 years old****]. "It is good and increases our*

confidence in performing procedures on patients." ***[4th year 21 years old].*** *Furthermore, OSCE was perceived as a method that comprehensively assesses clinical competence. For example, in this regard, an educator said: "... I think it is well organized and assesses them [their competence] very well."* ***[27 years old lecturer].***

All educators were satisfied with the exam format, its significant effect on teaching and learning, and the way it was designed. However, students' perceptions of the OSCE were somewhat affected by inadequate preparation time and resources, examiner interruptions, poor coordination, and a lack of feedback.

'[It is good; it has a good structure but lacks full coordination... time should be given adequately]'. ***[3rd-year student, 20 years old***]. *'Inadequate preparation for the examination and the involvement of examiners during the exam contributes to stress' (4th-year* ***student, 27 years old)***.

Implementation of OSCE by Respondents

Regarding the implementation of OSCE among midwifery and nursing educators, 43.5% (95%CI: 37.4, 49.5) had a good implementation. The results matched the findings of a qualitative study. Educators interviewed about the implementation of OSCE had the following responses. The implementation of OSCE status among participants varies according to their responses from 'good' to 'not well structured or standardized and low quality.

'To tell the truth, the implementation of OSCE becomes problematic once you have a large class [large number of students]. I didn't clearly understand how to use it as a

*teaching method, and it became difficult to get the appropriate ratio of teacher to student to give them timely feedback. ' (**31-year lecturer**).*

*'Good, but there was a shortage of instruments, or even if they were present, but they were not functional, this differs from a real patient scenario." (**34-year assistant professor).***

*'We apply it [OSCE], even if it is not standardized. Because a lack of resources, training, time constraints, and a large number of students per class can affect the implementation. Station preparation, time allocation, feedback, orientation provision, and scoring were varied accordingly '[32 years **old, lecturer]'***

The general organization of the OSCE was rated as equivocal by both the students and the educators. However, the students responded that the preparation and organization of OSCE needed to be enhanced. In addition to not allocating enough time for each task, some problems with OSCE organization include unclear instructions and distracting or "shouting" examiners.

Additionally, the students felt that the oral narration of the case scenarios caused bias and confusion before the task, and they perceived that the written case scenarios provided greater clarity than the oral narration.

*'While overall it was good, some of the instructions were not clear, and it would be better to make them clear. Some of the examiners were shouting.' **[3rd-year student, 23 years old].***

Moreover, students complained during in-depth interviews that they were not satisfied with the orientation provided, and they suggested that to familiarize themselves with the method of assessment, there had to be an orientation and practice OSCE session before the real exam.

'Most of us do not know how and what to prepare. We don't know the resources to read about preparation [for OSCE] ' [4th-year ***student, 25 years old]****. 'It is better if we practice it before.' [****4th year student, 22 years old****].*

Educators agreed that all learning domains were covered by the checklist and that each item only represented one topic. 147 (56.5%) agreed and strongly agreed that the checklist items were appropriate and in line with the time allotted and the specified learning objectives. However, only a considerable number of them, 86 (33.1%), expressed their experience that they had used a predetermined scoring checklist, and 161 (61.9%) educators agreed that timely individualized feedback was offered on test performance. The educators stated in an in-depth interview that

'I didn't develop a checklist; I just added some items or questions to the MOH checklist' [30-year-old ***lecturer****]. 'It would be difficult to reach all domains of competence in the absence of a trained simulator, especially communicative skills'.* ***[32-year-old lecturer****]. 'We do it [OSCE] by attempting to focus all knowledge, skill, and attitude, with great limitations due to different inconveniences.' [****27-year-old, lecturer****].*

Of the educators, only 97 (37.3%) agreed that 5-10 stations were used; only 117 (45%) agreed that the names and objectives of the stations were clearly described and in line with the course objectives. The number of stations in the sampled competencies was criticized by both educators and students. Maintaining a fair representation of competencies at OSCE stations was found to be a challenge for faculty.

One of the educators mentioned that it would be difficult even to imagine a representation of obstetric competence for 4th-year students only with two or three procedures such as normal labor management, AMTSL, and neonatal resuscitation.

'The topics covered are very important in obstetrics, and they are very common procedures…but [only] a limited number of stations were sampled.' [4th ***year student, 25 years old]***

Concerning time allocation, only 92 (35.4%) of the teachers agreed that 5–10 minutes were allowed per station. Additionally, key informants reported inadequate time to complete the stations. The students complained that there was not enough time allocated to each station to complete the given task completely. In this regard, some examiners concurred as well. They felt that even with fewer stations, there wasn't enough time allotted for each station.

"Time was not enough for the stations. I think it does not give us time to think and gather our thoughts. It's not fair. Even we did not have time to think.' ***[3rd year, 23 years old]*** *'It is better to have a sufficient number of stations with enough time'.* ***[4th year, 21 years].***

'With scoring and time allocation, there is not much new or groundbreaking. It all depends on the number of teachers and students involved in the course. ' ***[34 years old, assistant professor].***

Factors associated with OSCE implementation among respondents

In the multivariate logistic regression analysis, training on OSCE, availability of equipment, OSCE implementation checklist, and teacher perceptions were found to be significantly associated with OSCE implementation. Educators who had received OSCE training were twice as likely to implement OSCE compared to those who did not receive training (AOR = 2.070, %CI: 1.029, 4.171, p = 0.042).

The odds of OSCE implementation among educators who have the necessary equipment in their skill laboratory were 2.36 times higher than those who do not have the necessary equipment (AOR = 2.36, 95% CI 1.032, 5.395, p = 0.041). Similarly, educators who had an OSCE implementation checklist in their department were two times more likely than their counterparts (AOR = 1.893, 95% CI: 1.052, 3.420, p = 0.037).

Having a positive perception of OSCE had a statistically significant relationship with the implementation of OSCE, and those who had negative perceptions about OSCE were 46.8% less likely to implement OSCE than those who had positive perceptions of OSCE. (AOR = 0.532, 95% CI 0.307, 0.921, P = 0.024) (**Table 4)**

During an in-depth interview, the investigator tried to address possible barriers at each respective university. Most key informants claim that there is no single responsible

body for poor OSCE implementation. It requires the collaboration of all the entities involved at universities.

Every stakeholder has a significant and irreplaceable role in teaching and assessing the clinical performance of students. Major stakeholders raised by the discussants are the government, educators, technical assistants, and university management bodies, including the concerned nongovernmental organizations. Without coordinating the efforts of all these stakeholders, it is still difficult to perform in the expected way.

They also raised issues related to poor planning and coordination regarding schedules for practice and OSCE exams, a lack of standardization (nationally harmonized guideline), large numbers of students per class, and the negligence of educators to update existing checklists or develop new ones at the departmental level.

Another idea proclaimed by almost half of the participants focuses on the fact that poor orientation of students can stem from a lack of experience and training (a shortage of trained human power and a lower quality of training), a shortage of resources such as equipment, problems in the exam hall which leads to selecting insufficient number station, and the problem of time constraints.

Discussion

This study revealed that only 43.5% (95% CI: 37.4, 49.5) of educators had a good OSCE implementation. Training about OSCE, having an OSCE checklist, the availability of necessary equipment, and positive perceptions towards OSCE were significantly associated factors with OSCE implementation. Perceptions towards OSCE and its implementation, poor organization and planning, shortage of resources, and insufficient number and duration of stations were the major findings.

According to this study, only 43.5% (95% CI 37.4, 49.5) of the educators had a good OSCE implementation. The results of a qualitative study support this finding. The study's main promising findings were the positive perceptions of educators and students of the OSCE format. Student and examiner satisfaction with OSCE, the main reason for their preference was that OSCE truly evaluates their clinical skills and has more validity and reliability.

Despite the positive perceptions towards OSCE formats that encourage the implementation of OSCE in the future, the implementation of OSCE lacks several crucial elements such as adequate instruction and feedback, sufficient number and duration of stations, proper planning and preparation for OSCE, and the appropriate scoring that this study revealed.

This study suggests that the OSCE implementation process lacks proper organization and planning. Poor organization is a finding related to the failure to plan adequately

for the OSCE exam. During the planning phase, the OSCE team should schedule meetings and training sessions before the exam day to prepare the exam blueprint, design stations, develop scenarios, orient examiners, set up rooms and equipment, and handle other management concerns.

Educators and students both raised complaints about issues that were either directly or indirectly attributed to poor organization. Interfering educators, uncertain instructions, inadequate station and time allocation, and lack of materials were among the issues mentioned in the reports. Khan et al. proposed sharing responsibility among the team, exam schedule preparation, rules and regulations, preparation of the exam blueprint, determination of the station number and duration of the exam, development of the intended OSCE stations, preparation of the marking tool, and, lastly, piloting of the circuits developed before the OSCE exam to handle poor organization and related issues [25] .

Generally, the findings show that the implementation of OSCE is not being applied properly. This limits the interaction between patients and students during their clinical integration and their acquisition of the competencies required by the competency-based curriculum implementation approach.

This suggests that more than half of educators do not implement OSCE according to the OSCE quality assurance criteria in the exam implementation guide of the Minister of Health of Ethiopia [7] , this might be due to the short duration of time since its (OSCE) implementation started, and it needs time to scale up, create positive perceptions among educators and collaboration from all concerned bodies.

In addition, the narrow time gap between this study and the amendment of the exam manual could be a significant reason why the introduction of OSCE implementation at the Ethiopian Public University has recently started.

The findings of this study revealed that OSCE training was significantly associated with its implementation. Educators who had been trained were twice as likely to implement OSCE than those who had not been trained.

This finding is supported by the findings of the qualitative study. During in-depth interviews, from the examiner's side, the challenge of planning and preparation for OSCE was mainly perceived as related to logistics and the capacity building of the examiners. Training is essential to run OSCE, however, educators complained that the majority of the staff received any kind of training on OSCE, which can contribute to lowering its OSCE quality.

This finding was well in agreement with previous studies conducted in Addis Ababa, St. Paul’s Millennium Medical College, and the University of Caliber, Nigeria [17,22]. This could be because attending training can help educators gain more understanding of how to prepare the case scenario and run OSCE during the exam, allowing them to better implement. It can also lead to better standardization of the exam scoring [10]. This highlights the need for examiner training.

The result of this study revealed statistical significance between the implementation of OSCE by educators and the existence of a checklist; those who have a checklist implemented are two times more likely than their counterparts. Educators at IDI

complained that they encountered problems in implementing OSCE due to a lack of harmonized standard guidelines and recommended that they be prepared at the national level. It makes it easy to implement OSCE and helps to assess students objectively.

This is consistent with the qualitative studies conducted at Jimma, the Caliber University of Nigeria, and Addis Ababa Paul's Medical College [16,22,23]. It might be that the guideline guides educators in how, when, and which case scenarios should be selected for the exam; it also helps educators build confidence and increase their readiness to implement OSCE.

Another factor statistically related to the implementation of OSCE by educators was the availability of the necessary equipment. Those who have the necessary equipment in the skill lab were 2.3 times more likely to implement it compared to those who do not have equipment in the skill lab. Qualitative results complement this as well.

During the in-depth interview, almost all educators and students complained that the absence of necessary and functional equipment at the lab could affect the OSCE implementation. It limited the educator's ability to select a representative sample; practicing with assumptions cannot help students develop clinical skills.

Most educators and students revealed that there was a shortage of instruments, or even if they were present, they were not functional; this differs from a real patient scenario and affects the clinical skill development of the students. It reduces the quality of OSCE implementation.

An even higher percentage reported it as a challenge previously in studies done in India, Pakistan, Riyadh University of Saudi Arabia, and Malaysia [9,16,22,23,26] , This might be because OSCE is a very demanding examination method, requiring an abundant amount of human and material resources [22] .

Universities must make the availability of simulation materials a priority. A well-organized and complete simulation lab should be prepared both for practice and for final assessment.

Similarly, respondents' perceptions were significantly associated with their implementation of OSCE; those educators with a negative perception towards the OSCE, implementing OSCE were 46.8% less likely compared with those with a positive perception.

This is supported by studies from Pakistan, which report that those who have positive perceptions towards OSCE recommend that it be used in the future and would like it to apply to all batches, both by students and examiners. This may be because educators will feel more motivated to apply the OSCE if they have positive perceptions [23].

Implications of the Study

In general, the results of the current study indicate that less than half of teachers have a good OSCE implementation status, highlighting the need for improved training, support, and resource allocation for effective medical education. Ethiopian public universities in the central and southern regions should address gaps in OSCE

implementation by allocating resources and providing effective training for educators. This will improve the quality of healthcare education and ensure fair assessments, leading to better learning outcomes. The study findings can inform policy design, standardized guidelines, clear expectations, and regular evaluations.

Strengths and limitations of the study

The study was used at the regional level and incorporated mixed methods of data collection, including qualitative data from educators and students, to understand OSCE implementations and challenges, enhancing the credibility of the findings. However, the study did not use observational methods to observe educators' implementation of OSCE, potentially leading to social desirability bias.

Conclusion

The study found that less than half of the south and central Ethiopian university teachers had a good OSCE implementation, factors such as training, equipment availability, and educators' perceptions affecting their implementation. Issues such as poor organization, planning, resource scarcity, and insufficient number and duration of stations were identified as challenges in the OSCE implementation through a qualitative study.

Strengthening teacher training on OSCE implementation in Ethiopian universities, focusing on standard procedures, checklists, and feedback. The university administration should monitor the availability of equipment and help educators organize the OSCE hall for a quiet, distraction-free environment expected from

university stakeholders. Midwifery and nursing educators must be familiar with institution policies and implement OSCE with proper planning and organization. Validity should be ensured by focusing on the curriculum, important procedures, and clear instructions. Staying up-to-date on research and best practices ensures effective evaluation methods. Researchers should use observational methods to assess the implementation status and difficulties faced by educators.

Ethics Approval

This study has been approved by the Jimma University Institutional Review Board (Ref. number JUIH/IRB-345/2023). Jimma University Institute of Health wrote a letter of permission to each university, and before starting actual data collection, institution permission was obtained from all universities. An information sheet was prepared and explained to study participants that included the purpose and importance of the study, the confidentiality of the participant's response, and the benefits and risks of the study. Informed, voluntary, written, and signed consent was sought from the participants. The confidentiality of the data was guaranteed by using identification numbers rather than names and limiting access to the data.

Data availability section

All data available on manuscript

Acknowledgments

The authors thank the study participants, supervisors, and data collectors.

Authors' Contributions

All authors made a significant contribution to the reported work, whether that is in the conception, design of the study, execution, acquisition of data, analysis, and interpretation, or all these areas; took part in the drafting, reviewing, or critically reviewing the article; gave final approval of the version to be published; have agreed on the journal to which the article has been submitted; and agreed to be responsible for all aspects of the work.

Funding

This research work was funded by Jimma University. The sponsors had no role in study design, data collection, and analysis, decision to publish, or manuscript preparation.

Disclosure

The authors report that there are no conflicts of interest in this work.

References

1. Alizadeh S, Zamanzadeh V, Johnston A, Valizadeh L, Ghaffari R, Ayenew E, et al. Implementation of an OSCE at Kaohsiung Medical University. Suparyanto dan Rosad (2015 [Internet]. 2022;10(1):174–80. Available from: http://dx.doi.org/10.1186/s41077-016-0014-1

2. British T, Journal M. MEDICAL PRACTICE Medical Education. 2023;2(5910):99–107.

3. Khan KZ, Ramachandran S, Gaunt K. (OSCE): AMEE Guide No. 81 . Part I : An historical and theoretical perspective. 2013;(81).

4. Examination S. Medical Education Assessment of Clinical Competence using Objective Structured Examination. 1975;(February):447–51.

5. Shumway JM, Harden RM. The assessment of learning outcomes for the competent and reflective.

6. Choudhari S. Objective structured Clinical Examination (OSCE): Objective Structured Clinical Examination (OSCE) : A Review. 2021;(August 2016).

7. MoH F. Federal Democratic Republic of Ethiopia Ministry of Health Health Professionals' Competency Assessment and Licensure Directorate. 2019;(March).

8. Mamatha SD, Kanyakumari DH. Objective structured practical examination / objective structured clinical examination as an assessment tool : Faculty perception. 2018;8(11):2–5.

9. Peeters MJ, Cor MK, Petite SE, Schroeder MN. Note Validation Evidence using Generalizability Theory for an Objective Structured Clinical Examination. 2021;12(1):1–5.

10. Elshama SS. How to Design and Apply an Objective Structured Clinical Examination (OSCE) in Medical Education ? 2021;01:51–5.

11. E. Omu F. Attitudes of Nursing Faculty Members and Graduates towards the Objective Structured Clinical Examination (OSCE). Open J Nurs. 2016;06(05):353–64.

12. Ho C, Lin C, Chung U. Journal of Nursing & Care Short Communication A Short Commentary about Benefits and Drawbacks of Osces in the Nursing Education. 2015;5(1):1–2.

13. Ngim CF, Fullerton PD, Dominic NA. Feedback after OSCE : A comparison of face-to-face versus enhanced written feedback. 2020;1–16.

14. Blamoun J, Hakemi A, Armstead T. A guide for medical students and residents preparing for formative, summative, and virtual objective structured clinical examination (Osce):

Twenty tips and pointers. Adv Med Educ Pract. 2021;12:973–8.

15. Elfagi S, Elamni SF, Nouh F, Eltohami A, Omar M. Undergraduate Student Feedback on OSCE : The First Experience of a Nutrition Department, University of Benghazi 2019. 2020;2665:187–91.

16. Ataro G, Worku S, Asaminew T. Experience and Challenges of Objective Structured Clinical Examination (OSCE): Perspective of Students and Examiners in a Clinical Department of Ethiopian University. Ethiop J Health Sci. 2020;30(3):417–26.

17. D MMP, D EJP, D PP, Margaret C. Determinants Of OSCE Performance Among Nursing Students In Department Of Nursing Science, University Of Calabar, Calabar, Cross River State. 2022;6(3):6284–91.

18. Alkhateeb N, Salih AM, Shabila N, Al-Dabbagh A. Objective structured clinical examination: Challenges and opportunities from students' perspective. PLoS One [Internet]. 2022;17(9 September):1–9. Available from: http://dx.doi.org/10.1371/journal.pone.0274055

19. Alaskar A, Subbarayalu AV, Alfaraj E, Ramzi OI, Alameri NS, Alhababi A, et al. Health science students' perceptions about Objective Structured Clinical Examination (OSCE) as a method of clinical evaluation. Electron J Gen Med. 2022;19(6).

20. Shitu B, Girma T. OBJECTIVE STRUCTURED CLINICAL (OSCE): EXAMINEE ' S PERCEPTION AT DEPARTMENT OF PEDIATRICS AND CHILD HEALTH, JIMMA UNIVERSITY. (8):47–52.

21. Republic FD. Federal Democratic Republic of Ethiopia Ministry of Health exam administration Manual. 2015;6(1).

22. Fisseha H, Desalegn H. Perception of Students and Examiners about Objective Structured Clinical Examination in a Teaching Hospital in Ethiopia. Adv Med Educ Pract. 2021;12:1439–48.

23. Khan S, Khan S, Naz N, Khaum S, Zabeeda M, Siddique U, et al. Perception of Faculty Teachers Towards Objective Structured Clinical Evaluation in Public Nursing Colleges of Peshawar. 2022;157–61.

24. Braun V, Clarke V. Using thematic analysis in psychology. 2006;(January).

25. Khan KZ, Gaunt K, Ramachandran S, Pushkar P. The Objective Structured Clinical Examination (OSCE): AMEE Guide No. 81. Part II: Organisation & Administration. Med Teach. 2013;35(9):142–59.

26. Sallam A, Atwa H, Abdelaziz A, Nasser AA. Medical Students ' Perception of a Newly Implemented Objective Structured Clinical Examination (OSCE) in Orthopedic Surgery and Trauma : A Mixed-Method Study. 2022;22(March):29–38.

Tables

Table 1: Sociodemographic characteristics of educators who participated in the study and worked in universities in the south and central region, Ethiopia, 2023(N=260)

Variables	Category	Frequency	Percentage
Age in years	<25	23	8.8
	25-30	148	56.9

	31-35	54	20.8
	>35	35	13.5
Sex	Female	80	30.8
	Male	180	69.2
Religion	Protestant	118	45.4
	Orthodox	80	30.8
	Muslim	22	8.6
	Catholic	13	5.0
	*Other	28	10.8
Marital status	Single	100	38.5
	Married	152	58.5
	Divorced	8	3.1
Department	Midwifery	110	42.3
	Nursing	150	57.6
Job title/ academic rank	GA II	8	3.1
	Assistant lecturer	41	15.8
	Lecturer	156	60

	Assistant professor and above	55	21.2
Work experience in the year	1-5	140	53.8
	6-10	90	34.6
	Above 10	30	11.5
Training on OSCE	Yes	62	23.8
	No	198	76.2
Previous experience in OSCE preparation	Yes	164	63
	No	96	37

655 *Other=Apostolic, Jehovah Witness

656 ***Table 2: Generated themes and subthemes (code: 45, PDs*: 24 pages)***

S.No.	Themes	Categories or sub-themes
1	Perceptions towards OSCE and its implementations	• Level of implementation
		• Advantages of OSCE
		• Impact of OSCE on the teaching and learning process
2	Poor organization and planning	• Lack of planning and coordination
		• Poor orientation and feedback
		• Improper checklist preparation and use
3	Insufficient number and duration of the station	• Improper preparation of station
		• Shortage of time at the station and for preparation

4	Shortage of resources	• Shortage of instruments at the skill lab
		• Lack of training
		• Shortage of supportive staff
		• Unbalanced staff-to-student ratio Unbalanced staff ratio

**PD=primary document*

Table 3: OSCE facility-related factors among educators who participated in the study and worked in the Universities of the South and Central Region, Ethiopia, 2023 (N = 260).

Variables	Category	Frequency	Percentage
Checklist about OSCE at the department or skill lab	Yes	90	34.6
	No	170	65.4
OSCE is a continuous assessment method for core competency courses.	Yes	122	46.9
	No	138	53.1
#If no, what is the reason?	lack of resources	59	42.7
	Lack of training	40	28.9
	*Other	39	28.4
If OSCE was used for continuous assessment, timely feedback offered to students	Yes	69	56.6
	No	53	43.4

Having the OSCE exam hall, which occupies at least 5 station	Yes	94	36
	No	166	64
Availability necessary equipment	Yes	41	15.8
	No	219	84.2
Standardized patient or doll to simulate the Case scenario	Yes	102	39.2
	No	158	60.8
#multiple responses are possible, *Other= time consuming, staff shortage,			

Table 4: Bivariate and multivariate logistic regression analysis of factors associated with OSCE implementation among midwifery and nursing educators in universities of the south and central region, Ethiopia, 2023

Variables		Implementation of OSCE		COR (95%CI)	AOR (95% CI)	P-Value
		Good	Poor			
Gender	Female	39(15.0)	41(15.8)	1.362(1.002, 2.314)*	1.206(0.661, 2.200)	0.542
	Male	74(28.4)	106(40.8)	1	1	
Work	1-5	68(26.1)	72(27.7)	2.204(1.094, 5.147)*	1.820(0.692, 4.783)	0.225

Experience	6-10	36(13.8)	54(20.8)	1.556(0.640, 3.778)	1.216(0.466, 3.168)	0.690
	>10	9(3.5)	21(8.0)	1	1	
OSCE preparation experience	Yes	86(33.0)	78(30.0)	2.67(1.091, 5.551)*	2.773(0.929,7.470)	0.054
	No	28(10.8)	68(26.2)	1	1	
Training OSCE	Yes	34(13.0)	28(10.8)	1.829(1.029, 3.252)	2.070(1.029, 4.171)	0.042**
	No	79(30.4)	119(45.8)	1	1	
Availability of checklist	Yes	55(21.1)	35(13.5)	3.034(1.787, 5.152)	1.893(1.052, 3.420)	0.037**
	No	58(22.3)	112(43.1)	1	1	
having exam hall	Yes	54(20.9)	40(15.1)	2.488(1.478, 4.187)*	1.671(0.924, 3.521)	0.059
	No	59(22.9)	107(41.1)	1	1	
Availability of Equipment	Yes	28(10.8)	13(5.0)	3.395(1.666, 6.918)	2.360(1.032, 5.395)	0.041**
	No	85(32.7)	134(51.5)	1	1	
Perceptions	Negative	50(19.5)	88(34.4)	0.53(0.324, 0.874	0.532(0.307, 0.921)	0.024**
	Positive	63(23.8)	59(22.3)	1	1	

Note: * Statistically significant at P-value<0.25, ** at p<0.05, 95%CI, 1=Reference group

Abbreviation

AOR: Adjusted odd ratio

CI: Confidence interval

COR: Crude odd ratio

IDI: In-depth interview

JU: Jimma University

KI: Key informant

OSCE: Objective structured clinical examinations

OSPE: Objective structured practical examination

SP: Standardized patients
