# Supplementary material for: Implementation of objective structured clinical examination among midwifery and nursing educators at South and Central Region Universities in Ethiopia, 2023
Source: PLoS One. 2026 Aug 26;21(8):e0356551. doi: 10.1371/journal.pone.0356551 (PMC13514063; doi:10.1371/journal.pone.0356551)
Supplement: Supplementary file 2 [file pone.0356551.s004.pdf]

POINT-BY-POINT RESPONSE TO THE REVIEWER'S COMMENTS

5. Review Comments to the Author

Reviewer #1: The manuscript provides valuable insights into OSCE implementation challenges. However, improving clarity and adherence to standard English conventions will enhance its readability and impact. A thorough review focusing on simplifying language, ensuring consistency, and organizing content logically is recommended.

- ❖ We are grateful to the reviewer for succinctly pointing our attention to these grammatical errors, and consistency, organizing content logically. The entire manuscript has been revised to ensure clarity in expression avoid grammatical errors and organize logically.

Reviewer #2: For the introduction, please follow these steps:
1. Simplify Sentences: Break long sentences into shorter, clearer ones to improve readability. Remove repetitive phrases and redundant information.
2. Structure the Introduction: Start with a brief introduction to the OSCE, followed by its history, and then move into its implementation and challenges—group similar ideas together for better flow.
3. Consistent Terminology: Ensure consistent terminology and citation format throughout the text. Use either "OSCE" or "the OSCE" consistently.
4. Highlight Significance: Highlight the significance of the study and its unique contribution to the existing literature early on. Clearly state the theoretical framework that guided the study.
5. Grammar and Clarity: Correct grammatical errors and improve sentence structure. Avoid overly technical jargon where simpler terms can be used.

- ❖ This part is considered in the manuscript.

For method section
Please follow these steps.

1- Study Design Description: Provide a more detailed description of the mixed-methods approach, including how the qualitative and quantitative data were integrated.

- ❖ institution-based cross-sectional study was used that uses quantitative data supported by a qualitative study. which integrates both quantitative and qualitative data to provide a comprehensive understanding of the research problem. Triangulation of data was used, to enhance the validity of the findings.

2- Sampling Strategy: Clarify the rationale for the sample size and the selection of key informants for the qualitative component. Explain how the sample size ensures the representativeness of the findings.

- ❖ For the quantitative aspect, four universities (i.e., Wachemo, Wolkite, Arba Minch, and Wolaita Sodo universities) were randomly selected out of the six universities under the study area.
- ❖ All eligible educators were included in the research sample. Given the relatively small number of study participants, opting for a census method was practical and ensured comprehensive coverage.
- ❖ key informants (KI) was based on their years of experience and their positions within the school. specifically, to gain a more nuanced perspective, class representatives and students in their third year or higher were selected for in-depth interviews. The determination of the qualitative sample size followed the principle of data saturation, where interviews were conducted until the responses became repetitive, ensuring that the collected data were sufficient to address the study objectives in full.

3- Data Collection Tools: Provide more information on the validity and reliability of the data collection tools used. Mention any steps taken to pre-test or pilot these tools.

- ❖ Before the actual data collection; a pre-test was done with 10 % of midwifery and nursing educators and students in the central region, of Hosanna Health Science

College. Based on the findings from the pretest, some ambiguous questions and logical order were corrected.

- ❖ An ongoing formative checkup for completeness and consistency of responses was made by the data collectors daily. Data cleaning and reviewing for mistakes inconsistencies and adjustments were conducted to avoid errors in the labeling or order of the variables of interest. The collected data were reviewed, checked, coded, and cleaned for completeness.
- ❖ The overall standardized Cronbach's alpha for internal consistency or reliability score of OSCE implementation and perception measurement was 0.796 and 0.721 respectively.

4- Descriptive Statistics: Present demographic data of educators and students.

5- Comparative Analysis: Compare OSCE implementation and perceptions across different universities.

6- Correlation Analysis: Investigate correlations between experience and OSCE implementation scores.

7- Multivariate Analysis: Using logistic regression, identify factors associated with exemplary OSCE implementation.

8- Reliability and Validity: Validate your survey instrument with factor analysis and assess internal consistency with Cronbach's alpha.

9- Advanced Techniques: Consider SEM to explore relationships between multiple variables influencing OSCE implementation.

- ❖ Serial numbers 4 to 5 are considered in the manuscript part
  For results section

Tables: Ensure all tables (Table 1, Table 2, Table 3, and Table 4) are included and formatted correctly in the methods section. Each table should have a clear title and caption. Ensure the tables referenced in the text are presented in the results section. More Details: Provide a more detailed summary of the demographic data, including age distribution, academic rank distribution, and years of experience.

Edited.

• Themes and Subthemes: Clearly define and describe each theme and subtheme identified in the qualitative analysis. Provide more examples and explanations for each theme.

- ❖ Each theme and subthemes was identified and discussed.

• Detailed Reporting: Provide more details on the statistical tests used, such as the specific methods for logistic regression and the criteria for inclusion in the multivariate analysis.
• More details about the results of the instrument's reliability and validity.

Model Fit: Include information on model fit statistics for the logistic regression, such as the Hosmer-Lemeshow test results.

• Effect Sizes: Report effect sizes for significant findings to provide more context on the magnitude of the effects.

- ❖ The Hosmer-Lemeshow statistic was done for a model fitness test and was not significant (p=0.725) and the omnibus test was significant (p-value = 0.000) which indicates that the model was fitted.

Discussion:

Comparison with Literature: Compare your findings with existing literature more thoroughly. Discuss why specific findings might differ from those in other studies.
Implications for Practice: Based on the study's findings, provide more detailed recommendations for educators and policymakers. Discuss how the results can inform improvements in OSCE implementation.
Limitations: Discuss the study's limitations in more detail, including potential biases and the generalizability of the findings.

- ❖ This part is considered in the manuscript

Specific Comments on Sections

• Introduction (Lines 49-113): The historical background on OSCE is helpful but should be condensed to focus more on the specific challenges and needs in the Ethiopian context.

- ❖ We have included the following statements in lines 83- 107 of the revised manuscript to show specific challenges and needs in the revised manuscript.

• Methods (Lines 114-221): The description of the thematic analysis process is good but could benefit from a brief explanation of how themes were derived and validated.

• Results (Lines 222-422): The qualitative findings could be better integrated with the quantitative results to provide a more cohesive narrative.

- ❖ Thank you, This part is considered in the manuscript.

• Discussion (Lines 427-552): The discussion should more explicitly connect the findings to broader theoretical frameworks and practical implications.

- ❖ Considered in the manuscript part

- ❖ Thank you.
