# Supplementary material for: Implementation of objective structured clinical examination among midwifery and nursing educators at South and Central Region Universities in Ethiopia, 2023
Source: PLoS One. 2026 Aug 26;21(8):e0356551. doi: 10.1371/journal.pone.0356551 (PMC13514063; doi:10.1371/journal.pone.0356551)
Supplement: Supplementary file 3 [file pone.0356551.s003.docx]

Thank you for the opportunity to review this paper.

This manuscript explores an important issue regarding the implementation of Objective Structured Clinical Examinations (OSCEs) among midwifery and nursing educators in Ethiopia. The mixed-methods design is appropriate and contributes to a more comprehensive understanding of the barriers and facilitators in educational settings.

The topic is timely and relevant, especially as many low- and middle-income countries are increasingly integrating competency-based assessments into healthcare education. The integration of both quantitative and qualitative data provides useful insights into the contextual challenges faced in implementing OSCEs. The response rate is commendably high, and the triangulation approach enhances the credibility of the findings.

However, the manuscript would benefit from revisions that address several key areas, particularly the clarity of measurement, transparency of reliability testing, completeness of abbreviation use in tables, and a more in-depth discussion of systemic factors affecting implementation. Addressing these concerns would strengthen the manuscript’s contribution to the literature on educational assessment in health professions and improve its alignment with the standards expected in PLOS ONE.

I commend the authors for their work and hope that the suggested revisions will assist in refining the manuscript further.

1. Page 4, Introduction, last paragraph (Lines 105–113): The justification for the study is not sufficiently distinct from existing literature. While the authors emphasize a lack of data in Ethiopia, the novelty of the study compared to other LMICs is not clearly articulated. Strengthen the justification by explicitly stating what this study adds to existing OSCE implementation research, especially in the context of sub-Saharan Africa or LMICs.
2. Page 12, Measurement and Operational Definitions (Lines 195–214): The manuscript mentions Cronbach’s alpha calculation in the Quality Control section but does not present the actual values in the Measurement section where tool validity and scoring methods are described. Report the Cronbach’s alpha values for the OSCE implementation and perception scales in the Measurement section to demonstrate internal consistency.
3. Page 8, Methods – Sampling for qualitative data (Lines 144–147): The manuscript states that data saturation was used to determine the qualitative sample size, but the specific criteria or evidence for saturation are not described. Clearly define how data saturation was assessed (e.g., repetition of codes, no emergence of new themes) and how it guided final sample determination.
4. Pages 37–38, Table 1 and Table 2: Several abbreviations such as GA II, OSCE, and AOR are not defined within the tables or footnotes. OSCE is used extensively but never defined in table footnotes.
5. Pages 24–27, Discussion section (Lines 446–553): The discussion identifies barriers such as limited resources and negative perceptions but lacks deeper analysis of systemic, policy-related, or socio-cultural root causes. Expand the discussion by linking identified factors to broader contextual issues (e.g., healthcare training policy, faculty development, resource allocation in LMIC settings).
6. Page 28, Strengths and Limitations (Lines 554–559): The limitations section briefly mentions the lack of observational methods but does not sufficiently address potential response biases due to the use of self-administered questionnaires. Discuss social desirability bias as a potential limitation and suggest methodological alternatives or mitigations in future studies.
7. Page 28, Conclusion (Lines 560–576): While the conclusion summarizes the findings effectively, it lacks actionable and context-sensitive recommendations. Provide specific strategies for universities, such as national standardization of OSCE checklists, faculty development programs, or investment in simulation infrastructure. These would strengthen the practical utility of the study.
