# Supplementary material for: Implementation of objective structured clinical examination among midwifery and nursing educators at South and Central Region Universities in Ethiopia, 2023
Source: PLoS One. 2026 Aug 26;21(8):e0356551. doi: 10.1371/journal.pone.0356551 (PMC13514063; doi:10.1371/journal.pone.0356551)
Supplement: Supplementary file 4 [file pone.0356551.s005.docx]

Subject: Revised manuscript submission.

Dear Dr. Professor Christmal Dela Christmals,

Thank you for your constructive feedback on our manuscript. Based on the reviewers' valuable insights, we have completed the revisions to our manuscript.

Their comments were incredibly helpful in strengthening our work, and we appreciate the time and care they took in evaluating our study.

We've incorporated all suggested changes, including your recommendation about the title. The revised title is now “Objective Structured Clinical Examinations (OSCEs) Implementation among Midwifery and Nursing Educators in South and Central Region Universities, Ethiopia, 2023.” We agree that this version better captures the essence of our research.

The review process has significantly enhanced our paper, and we're eager to share this improved version with you. Thank you very much for your insightful and constructive feedback on our manuscript.

Sincerely,

Zerihun Demessie
