# Supplementary material for: Implementation of objective structured clinical examination among midwifery and nursing educators at South and Central Region Universities in Ethiopia, 2023
Source: PLoS One. 2026 Aug 26;21(8):e0356551. doi: 10.1371/journal.pone.0356551 (PMC13514063; doi:10.1371/journal.pone.0356551)
Supplement: Supplementary file 5 [file pone.0356551.s006.docx]

**Response to Reviewer 2 Comments**

"The authors have revised the manuscript based on my suggestions from the previous peer review. However, there are still some areas that need to be revised, but I forgot to point out the significant figures of the AORs and their 95% CIs in the previous peer review.

Please refer to the following authoritative source….

The significant figures of the odds ratios and their 95% CIs should be two decimal places.

Please correct the significant figures in the following parts:
In the text:
L35-38
L443-446
L450
and
Table 3"

- Thank you for your valuable feedback and comments on our manuscript. We have carefully reviewed your suggestions and have made the necessary corrections, including the significant figures of the adjusted odds ratios (AORs) and their 95% confidence intervals (CIs) to two decimal places, as you recommended.
- The revisions have been incorporated in the following sections:
- In the text: Lines 35-38, 443-446, and 450
- Table 3
- We appreciate your thorough review and guidance, which have greatly improved the quality of our manuscript. Thank you once again for your support
