# Supplementary material for: Implementation of objective structured clinical examination among midwifery and nursing educators at South and Central Region Universities in Ethiopia, 2023
Source: PLoS One. 2026 Aug 26;21(8):e0356551. doi: 10.1371/journal.pone.0356551 (PMC13514063; doi:10.1371/journal.pone.0356551)
Supplement: Supplementary file 6 [file pone.0356551.s007.docx]

**Point by point response to Reviewer #6**

**#1. Select keywords from the MeSH.**

Keywords Replaced keywords with MeSH terms

- Clinical Competence
- Education, Nursing
- Education, Midwifery
- Ethiopia

**2. Write a brief introduction and remove unnecessary content.**

- Thank you for your insightful feedback and for taking the time to review our manuscript. We appreciate your valuable contribution to our work, and your comments have significantly improved the clarity and accuracy of our paper. *We incorporated under introduction section*

**3. Please refer to the author's guideline for editing the manuscript.**

Thank you for your insightful feedback and for taking the time to review our manuscript. We appreciate your recommendation. We referred to the author's guidelines for editing the manuscript

**4. Is this a mixed-methods study?**

- Yes, explicitly state in Abstract and Methods.

**5. The study design is not clear. The research design could be a little more detailed and linked to a philosophical position in line with the aim of the study.**

**Thank you for your insightful comment and feedback, and we added** a sentence in Methods: like
*“The study followed a pragmatic research paradigm, using mixed methods to gain a comprehensive understanding of OSCE implementation practices.”*

**6. Improve your tables with academic formatting.**

**Thanks for the suggestion, we incorporated all feedback and comments under manuscript.**

- Remove grid lines removed
- Consistent decimal places used
- titles and footnotes added
- Alignment (left for text, center for numbers) ensured

**#7. The results section contained some interesting but rather critical quotes. These need to be written in the accepted convention.**

- *Incorporated under manuscript*

**8. What has been done to ensure rigor in this study? Please explain more.**

- *Rigor was ensured through:*
- *Triangulation (quantitative + qualitative)*
- *Pretesting and expert validation*
- *Reliability testing (Cronbach’s alpha)*
- *Data saturation in qualitative sampling*
- Peer debriefing and bracketing in qualitative analysis

**#9. Discuss more about limitations of the study.**

- We appreciate your valuable contribution to our work, and your comments have significantly improved the clarity and accuracy of our paper, we added the following points to Limitations:
- Social desirability bias in self-reported data
- Lack of observational data on actual OSCE conduct
- Regional focus may limit generalizability
- Cross-sectional design prevents causal inference

**10. Make sure the referencing structure conforms to the convention of the journal.**

- Thank you for your insightful feedback and for taking the time to review our manuscript.
- We Checked PLOS ONE reference style (Vancouver, numbered) and ensured all references are complete and URLs/DOIs are included where available.
- We appreciate your thorough review and guidance, which have greatly improved the quality of our manuscript. Thank you once again for your support
