# Supplementary material for: Implementation of objective structured clinical examination among midwifery and nursing educators at South and Central Region Universities in Ethiopia, 2023
Source: PLoS One. 2026 Aug 26;21(8):e0356551. doi: 10.1371/journal.pone.0356551 (PMC13514063; doi:10.1371/journal.pone.0356551)
Supplement: Supplementary file 7 [file pone.0356551.s008.docx]

**Response to Academic Editor– [PONE-D-24-18847R4]**

Dear Ayse Hilal Bati, Professor, Academic Editor, PLOS ONE

Thank you very much for your insightful and constructive feedback on our manuscript. We appreciate your recognition that the research report has become more effective through the editing stages and that it successfully addresses the core research objectives.

We have carefully reviewed your suggestions and have fully revised the manuscript to incorporate these vital details. Below is a detailed summary of how these specific findings have been incorporated into the revised manuscript:

**1. OSCE Implementation across Universities**

- Revision: We have expanded the Results sections to highlight statistically significant differences in OSCE implementation among the studied institutions ($P = 0.014$).
- Key Data Points Added: The text now highlights that Wolkite University had the highest rate of poor implementation (71.4%), whereas Wachemo University (54.1%) and Arbaminch University (50.7%) demonstrated the highest rates of good implementation.

**2. OSCE Implementation Differences Across Academic Rank and Experience Level**

- Revision: We have incorporated the stratified data regarding faculty demographics into the report to address your note on experience levels and ranks.
- Key Data Points Added:
  - Work Experience: While differences across experience levels were not statistically significant ($P = 0.188$), the descriptive data clearly show a trend: educators with >10 years of experience reported the highest percentage of poor implementation (70%).
  - Academic Rank: Similarly, the variation across academic ranks ($P = 0.435$) has been contextualized. We note that Graduate Assistant II and Assistant Lecturer cohorts showed slightly higher percentages of good implementation (62.5% and 51.2%, respectively) compared to Lecturers and Assistant Professors.

**3. OSCE Implementation across Departments**

- Revision: As requested, we have clearly defined how OSCE implementation behaves as a critical determinant across different faculties, explicitly breaking down the data by department ($P = 0.033$).
- Key Data Points Added: The manuscript now emphasizes that the Midwifery department achieved a higher rate of good OSCE implementation (50.9%) compared to the Nursing department, where poor implementation was more prevalent (62.3%).

We believe these additions have significantly strengthened the depth, clarity, and overall success of the research report. The revised manuscript with track changes [or as per guidelines] has been uploaded.

Thank you once again for your guidance in improving this work.

Sincerely,

**Zerihun Demessie / Corresponding Author**

Department of Midwifery, Hossana College of Health Science, Hossana, Ethiopia
