# Supplementary material for: Implementation of objective structured clinical examination among midwifery and nursing educators at South and Central Region Universities in Ethiopia, 2023
Source: PLoS One. 2026 Aug 26;21(8):e0356551. doi: 10.1371/journal.pone.0356551 (PMC13514063; doi:10.1371/journal.pone.0356551)
Supplement: Supplementary file 8 [file pone.0356551.s009.docx]

**Response to Academic Editor– [PONE-D-24-18847R4]**

Dear, Ayse Hilal Bati, Professor, Academic Editor, PLOS ONE

Thank you for your follow-up feedback on our manuscript: **[PONE-D-24-18847R4]**). We sincerely appreciate your guidance regarding the need for a clearer, actionable interpretation of our findings in the Discussion section.

In line with your suggestion, we have thoroughly revised this section to provide a clear interpretation of our findings, rather than simply restating the results. Specifically, we have incorporated possible explanations for the observed variations in OSCE implementation across departments, universities, and faculty experience/rank, supported by relevant literature. We have also added a recommendation encouraging future researchers to conduct comparative studies, as such comparisons were beyond the scope of our study objectives.

All new additions have been highlighted using [Track Changes] in the revised manuscript.

We believe these revisions have strengthened the manuscript and given the data greater meaning and applicability, as you kindly noted. We are grateful for your guidance throughout this process and for your consideration of our work for publication in PLOS ONE.

Please let us know if any further revisions are required.

Thank you once again for your time and valuable input.

Sincerely,

**Zerihun Demessie / Corresponding Author**

Department of Midwifery, Hossana College of Health Science, Hossana, Ethiopia.
