# Supplementary material for: Implementation of objective structured clinical examination among midwifery and nursing educators at South and Central Region Universities in Ethiopia, 2023
Source: PLoS One. 2026 Aug 26;21(8):e0356551. doi: 10.1371/journal.pone.0356551 (PMC13514063; doi:10.1371/journal.pone.0356551)
Supplement: Peer review history

# Peer review: Decision Letter 0

**DOI:** 10.1371/journal.pone.0356551.r001

**Authors:** Rano Piryani

28 May 2024

Dear Dr. Demessie,

Thank you for submitting your manuscript to PLOS ONE. After careful consideration, we feel that it has merit but does not fully meet PLOS ONE’s publication criteria as it currently stands. Therefore, we invite you to submit a revised version of the manuscript that addresses the points raised during the review process.

Please submit your revised manuscript by Jul 12 2024 11:59PM. If you will need more time than this to complete your revisions, please reply to this message or contact the journal office at plosone@plos.org. When you're ready to submit your revision, log on to https://www.editorialmanager.com/pone/ and select the 'Submissions Needing Revision' folder to locate your manuscript file.

- A rebuttal letter that responds to each point raised by the academic editor and reviewer(s). You should upload this letter as a separate file labeled 'Response to Reviewers'.
- A marked-up copy of your manuscript that highlights changes made to the original version. You should upload this as a separate file labeled 'Revised Manuscript with Track Changes'.
- An unmarked version of your revised paper without tracked changes. You should upload this as a separate file labeled 'Manuscript'.

If you would like to make changes to your financial disclosure, please include your updated statement in your cover letter. Guidelines for resubmitting your figure files are available below the reviewer comments at the end of this letter.

If applicable, we recommend that you deposit your laboratory protocols in protocols.io to enhance the reproducibility of your results. Protocols.io assigns your protocol its own identifier (DOI) so that it can be cited independently in the future. For instructions see: https://journals.plos.org/plosone/s/submission-guidelines#loc-laboratory-protocols. Additionally, PLOS ONE offers an option for publishing peer-reviewed Lab Protocol articles, which describe protocols hosted on protocols.io. Read more information on sharing protocols at https://plos.org/protocols?utm_medium=editorial-email&utm_source=authorletters&utm_campaign=protocols.

We look forward to receiving your revised manuscript.

Kind regards,

Rano Mal Piryani, MBBS, MCPS, DTCD, MD, Fellowship in Med Education

Academic Editor

PLOS ONE

Journal Requirements:

When submitting your revision, we need you to address these additional requirements.

1. Please ensure that your manuscript meets PLOS ONE's style requirements, including those for file naming. The PLOS ONE style templates can be found at

https://journals.plos.org/plosone/s/file?id=wjVg/PLOSOne_formatting_sample_main_body.pdf and

https://journals.plos.org/plosone/s/file?id=ba62/PLOSOne_formatting_sample_title_authors_affiliations.pdf

2. We note that the grant information you provided in the ‘Funding Information’ and ‘Financial Disclosure’ sections do not match.

When you resubmit, please ensure that you provide the correct grant numbers for the awards you received for your study in the ‘Funding Information’ section.

3. Please note that funding information should not appear in the Acknowledgments section or other areas of your manuscript. We will only publish funding information present in the Funding Statement section of the online submission form. Please remove any funding-related text from the manuscript.

4. Please provide a complete Data Availability Statement in the submission form, ensuring you include all necessary access information or a reason for why you are unable to make your data freely accessible. If your research concerns only data provided within your submission, please write "All data are in the manuscript and/or supporting information files" as your Data Availability Statement.

5. When completing the data availability statement of the submission form, you indicated that you will make your data available on acceptance. We strongly recommend all authors decide on a data sharing plan before acceptance, as the process can be lengthy and hold up publication timelines. Please note that, though access restrictions are acceptable now, your entire data will need to be made freely accessible if your manuscript is accepted for publication. This policy applies to all data except where public deposition would breach compliance with the protocol approved by your research ethics board. If you are unable to adhere to our open data policy, please kindly revise your statement to explain your reasoning and we will seek the editor's input on an exemption. Please be assured that, once you have provided your new statement, the assessment of your exemption will not hold up the peer review process.

**Additional Editor Comments:**

Dear Authors

Greetings of the day

You have put your sincere efforts in doing this study and writing manuscript. You are advised to revise the manuscript in the light of reviewers comments.

[Note: HTML markup is below. Please do not edit.]

Reviewers' comments:

Reviewer's Responses to Questions

**Comments to the Author**

1. Is the manuscript technically sound, and do the data support the conclusions?

Reviewer #1: No

Reviewer #2: Yes

**********

2. Has the statistical analysis been performed appropriately and rigorously?

Reviewer #1: No

Reviewer #2: Yes

**********

3. Have the authors made all data underlying the findings in their manuscript fully available?

The PLOS Data policy

Reviewer #1: Yes

Reviewer #2: Yes

**********

4. Is the manuscript presented in an intelligible fashion and written in standard English?

Reviewer #1: No

Reviewer #2: Yes

**********

Reviewer #1: The manuscript provides valuable insights into OSCE implementation challenges. However, improving clarity and adherence to standard English conventions will enhance its readability and impact. A thorough review focusing on simplifying language, ensuring consistency, and organizing content logically is recommended.

Reviewer #2: For the introduction, please follow these steps:

1. Simplify Sentences: Break long sentences into shorter, clearer ones to improve readability. Remove repetitive phrases and redundant information.

2. Structure the Introduction: Start with a brief introduction to the OSCE, followed by its history, and then move into its implementation and challenges—group similar ideas together for better flow.

3. Consistent Terminology: Ensure consistent terminology and citation format throughout the text. Use either "OSCE" or "the OSCE" consistently.

4. Highlight Significance: Highlight the significance of the study and its unique contribution to the existing literature early on. Clearly state the theoretical framework that guided the study.

5. Grammar and Clarity: Correct grammatical errors and improve sentence structure. Avoid overly technical jargon where simpler terms can be used.

For method section

Please follow these steps.

1- Study Design Description: Provide a more detailed description of the mixed-methods approach, including how the qualitative and quantitative data were integrated.

2- Sampling Strategy: Clarify the rationale for the sample size and the selection of key informants for the qualitative component. Explain how the sample size ensures the representativeness of the findings.

3- Data Collection Tools: Provide more information on the validity and reliability of the data collection tools used. Mention any steps taken to pre-test or pilot these tools.

4- Descriptive Statistics: Present demographic data of educators and students.

5- Comparative Analysis: Compare OSCE implementation and perceptions across different universities.

6- Correlation Analysis: Investigate correlations between experience and OSCE implementation scores.

7- Multivariate Analysis: Using logistic regression, identify factors associated with exemplary OSCE implementation.

8- Reliability and Validity: Validate your survey instrument with factor analysis and assess internal consistency with Cronbach's alpha.

9- Advanced Techniques: Consider SEM to explore relationships between multiple variables influencing OSCE implementation.

For results section

• Tables: Ensure all tables (Table 1, Table 2, Table 3, and Table 4) are included and formatted correctly in the methods section. Each table should have a clear title and caption. Ensure the tables referenced in the text are presented in the results section. More Details: Provide a more detailed summary of the demographic data, including age distribution, academic rank distribution, and years of experience.

• Themes and Subthemes: Clearly define and describe each theme and subtheme identified in the qualitative analysis. Provide more examples and explanations for each theme.

• Detailed Reporting: Provide more details on the statistical tests used, such as the specific methods for logistic regression and the criteria for inclusion in the multivariate analysis.

• More details about the results of the instrument's reliability and validity.

• Model Fit: Include information on model fit statistics for the logistic regression, such as the Hosmer-Lemeshow test results.

• Effect Sizes: Report effect sizes for significant findings to provide more context on the magnitude of the effects.

Discussion:

Comparison with Literature: Compare your findings with existing literature more thoroughly. Discuss why specific findings might differ from those in other studies.

Implications for Practice: Based on the study's findings, provide more detailed recommendations for educators and policymakers. Discuss how the results can inform improvements in OSCE implementation.

Limitations: Discuss the study's limitations in more detail, including potential biases and the generalizability of the findings.

Specific Comments on Sections

• Introduction (Lines 49-113): The historical background on OSCE is helpful but should be condensed to focus more on the specific challenges and needs in the Ethiopian context.

• Methods (Lines 114-221): The description of the thematic analysis process is good but could benefit from a brief explanation of how themes were derived and validated.

• Results (Lines 222-422): The qualitative findings could be better integrated with the quantitative results to provide a more cohesive narrative.

• Discussion (Lines 427-552): The discussion should more explicitly connect the findings to broader theoretical frameworks and practical implications.

**********

what does this mean?). If published, this will include your full peer review and any attached files.

If you choose “no”, your identity will remain anonymous but your review may still be made public.

**Do you want your identity to be public for this peer review?**  For information about this choice, including consent withdrawal, please see our Privacy Policy

Reviewer #1: No

Reviewer #2: **Yes:** Eman S. Alsatari

**********

[NOTE: If reviewer comments were submitted as an attachment file, they will be attached to this email and accessible via the submission site. Please log into your account, locate the manuscript record, and check for the action link "View Attachments". If this link does not appear, there are no attachment files.]

While revising your submission, please upload your figure files to the Preflight Analysis and Conversion Engine (PACE) digital diagnostic tool, https://pacev2.apexcovantage.com/. PACE helps ensure that figures meet PLOS requirements. To use PACE, you must first register as a user. Registration is free. Then, login and navigate to the UPLOAD tab, where you will find detailed instructions on how to use the tool. If you encounter any issues or have any questions when using PACE, please email PLOS at figures@plos.org. Please note that Supporting Information files do not need this step.

Attachment Submitted filename: PONE-D-24-18847_review.pdf

# Author comment: Author response to Decision Letter 1

**DOI:** 10.1371/journal.pone.0356551.r002

16 Jan 2025

yes, I reviewed it

Attachment Submitted filename: point by point response to reviewers comments.pdf

# Peer review: Decision Letter 1

**DOI:** 10.1371/journal.pone.0356551.r003

**Authors:** Christmal Christmals

16 Apr 2025

Dear Dr. Demessie,

Thank you for submitting your manuscript to PLOS ONE. After careful consideration, we feel that it has merit but does not fully meet PLOS ONE’s publication criteria as it currently stands. Therefore, we invite you to submit a revised version of the manuscript that addresses the points raised during the review process.

Please submit your revised manuscript by May 31 2025 11:59PM. If you will need more time than this to complete your revisions, please reply to this message or contact the journal office at plosone@plos.org. When you're ready to submit your revision, log on to https://www.editorialmanager.com/pone/ and select the 'Submissions Needing Revision' folder to locate your manuscript file.

- A rebuttal letter that responds to each point raised by the academic editor and reviewer(s). You should upload this letter as a separate file labeled 'Response to Reviewers'.
- A marked-up copy of your manuscript that highlights changes made to the original version. You should upload this as a separate file labeled 'Revised Manuscript with Track Changes'.
- An unmarked version of your revised paper without tracked changes. You should upload this as a separate file labeled 'Manuscript'.

If you would like to make changes to your financial disclosure, please include your updated statement in your cover letter. Guidelines for resubmitting your figure files are available below the reviewer comments at the end of this letter.

If applicable, we recommend that you deposit your laboratory protocols in protocols.io to enhance the reproducibility of your results. Protocols.io assigns your protocol its own identifier (DOI) so that it can be cited independently in the future. For instructions see: https://journals.plos.org/plosone/s/submission-guidelines#loc-laboratory-protocols. Additionally, PLOS ONE offers an option for publishing peer-reviewed Lab Protocol articles, which describe protocols hosted on protocols.io. Read more information on sharing protocols at https://plos.org/protocols?utm_medium=editorial-email&utm_source=authorletters&utm_campaign=protocols.

We look forward to receiving your revised manuscript.

Kind regards,

Christmal Dela Christmals, PhD, MSc, BSc, RN

Academic Editor

PLOS ONE

Additional Editor Comments:

Dear Author,

The reviewers' decisions regarding the study were diverse.

We have provided several comments to enhance your study. I will be glad if you can revise the paper according to the guidance provided in the reviewer comments.

I would advise you to remove the phrase "and associated factors" from the title of the study.

Sincerely,

Professor Christmal Dela Christmals

[Note: HTML markup is below. Please do not edit.]

Reviewers' comments:

Reviewer's Responses to Questions

**Comments to the Author**

Reviewer #1: All comments have been addressed

Reviewer #3: (No Response)

Reviewer #4: (No Response)

**********

2. Is the manuscript technically sound, and do the data support the conclusions?

Reviewer #1: Partly

Reviewer #3: No

Reviewer #4: Yes

**********

3. Has the statistical analysis been performed appropriately and rigorously?

Reviewer #1: Yes

Reviewer #3: No

Reviewer #4: Yes

**********

4. Have the authors made all data underlying the findings in their manuscript fully available?

The PLOS Data policy

Reviewer #1: Yes

Reviewer #3: No

Reviewer #4: Yes

**********

5. Is the manuscript presented in an intelligible fashion and written in standard English?

Reviewer #1: Yes

Reviewer #3: No

Reviewer #4: Yes

**********

Reviewer #1: (No Response)

Reviewer #3: Implementation of Objective Structured Clinical Examination and Associated Factors among Midwifery and Nursing Educators at South and Central Region Universities in Ethiopia, 2023

PLOS ONE

PONE-D-24-18847R1

Thank you for inviting me to review the above-titled manuscript. The topic may be interesting to education in Ethiopia. However, the manuscript has significant problems in the study design, the methods used, the way it was presented and the validity and reliability of tools and methods. Also, there is no educational basis for the study. It is not suitable for PLOS ONE or the broader readership. The aim of the study has been in many publications in English Literature over 25 years ago.

TITLE: NOT CLEAR. WHAT DO YOU MEAN BY ASSOCIATED FACTORS? What is the purpose of the study other than implementation?

ABSTRACT: 1) Statements (lines 23/25) are not accurate. There are many studies answering this aim. 2) What is the research question? 3) What do you mean by associated factors? Repeated several times. 4) How many universities (line 30)? How were they randomly selected? 5) Sources of the Questionnaire? Validity and reliability measures? Missing. 6) Who was invited for the interviews? How selected? How conducted? Not clear even in the study body 7) What do you mean by "GOOD IMPLEMENTATIONS"? What are the standards? Sources of these standards? How did you measure things to come up with this conclusion? 8) Nothing new in the study. We know these answers many years ago. There are even books written on OSCE that answer all these basics. Read Academic Medicine, Medical Education, Medical Teacher, BMC Medical Education, Teaching and Learning in Medicine, Advances in Health Sciences Education and others.

INTRODUCTION- 1) Mostly copied. It should be focused. We need to know what triggered the study. 2) Discuss what the literature has answered and identify real gaps. Your references reveal that you are not aware of the proper literature in this area. 3) What is the research question? 4) Why do we need this study? What is new? 5) What gaps? Is it in preparation for OSCE, in training OSCE examiners, in training students to answer OSCE, in preparation for OSCE stations, in marking OSCE tests, or must you be clear and focused?

METHODS: 1) Poorly written and missing important information. 2) Methods need references/citations. 3) What is the sample size? How was it calculated? Show equation. 4) Male and Females in your sample. Why do 180 males and 80 only females? in Midwifery look strange? What is the culture in Ethiopia? 5) Questionnaire: Why was it adapted from Ref #7, 16, 17, 20, 21, and 22? Justify why you did not use a questionnaire from the literature. 6) How the participants were selected? 7) What are the validity and reliability measures of the questionnaire? Missing. 8) The date of ethical approval is missing. 9) What are the statistics used? How used? Purpose? add a citation. 10) How the participants in interviews were selected? Numbers, and how were these interviews carried out? 11) The study should be piloted.

EDUCATION Basis is missing from the study introduction, methods, and discussion.

RESULTS- 1) Male and Females in your sample. Why do 180 males and 80 only females? in Midwifery look strange? What is the culture in Ethiopia? 2) In Table 3, the numbers were changed to males 74 and females 39. You need to explain why? 3) THere are other problems in methods concerning validity and reliability of the questionnaire. 4) NOTHING NEW

DISCUSSION AND CONCLUSIONS- This is not the standard for papers submitted for peer review. There are several biases in the study design, and the method used and tools. Also Educational Grounds are missing. Please read the literature.

REFERENCES: 1) Reference 4 is incomplete (which Journal), 2) References 5, and 6 are also incomplete. 3) Reference #7 add the URL. 4) References 8, 9, 10, 16, 28 which joiurnals published these studies? 5) Crucial studies were left out and unrelated or peripheral studies were cited. 6) Read comments in methods. 6) How the references you mentioned 7, 16, 17, 20, 21, 22 were cited for your questionnaire? Do these references have a questionnaire? No. What was the process of developing and testing a questionnaire? Read how we write a reference. Read the journal guidelines.

Reviewer #4: Evidence-based medical education is essential to establish better medical care. The purpose of this paper is assessment of OSCE implementation and associated factors among midwifery and nursing educators in the authors' country.

The study design and statistical analyses were generally performed appropriately, and the conclusions are considered to be valid.

Some incorrect terminology needs to be corrected: The authors use incorrect terms, i.e., "adjusted odd ratio" and "crude odd ratio". Correct them to "adjusted odds ratio" and "crude odds ratio".

**********

what does this mean?). If published, this will include your full peer review and any attached files.

If you choose “no”, your identity will remain anonymous but your review may still be made public.

**Do you want your identity to be public for this peer review?**  For information about this choice, including consent withdrawal, please see our Privacy Policy

Reviewer #1: **Yes:** Hye Won Jeong

Reviewer #3: **Yes:** PROFESSOR SAMY A AZER

Reviewer #4: **Yes:** Ryuichiro ARAKI

**********

[NOTE: If reviewer comments were submitted as an attachment file, they will be attached to this email and accessible via the submission site. Please log into your account, locate the manuscript record, and check for the action link "View Attachments". If this link does not appear, there are no attachment files.]

While revising your submission, please upload your figure files to the Preflight Analysis and Conversion Engine (PACE) digital diagnostic tool, https://pacev2.apexcovantage.com/. PACE helps ensure that figures meet PLOS requirements. To use PACE, you must first register as a user. Registration is free. Then, login and navigate to the UPLOAD tab, where you will find detailed instructions on how to use the tool. If you encounter any issues or have any questions when using PACE, please email PLOS at figures@plos.org. Please note that Supporting Information files do not need this step.

Attachment Submitted filename: PLOS ONE_review.docx

# Author comment: Author response to Decision Letter 2

**DOI:** 10.1371/journal.pone.0356551.r004

27 May 2025

Thank you for your thorough and insightful review of our manuscript. We greatly appreciate the detailed feedback, which has helped us identify key areas for improvement. Your suggestions align with our goal of enhancing the clarity, depth, and practical utility of the study. on the response, we address each of your points.

We believe these revisions will address your concerns and enhance the overall quality of the work. We are happy to make any further adjustments based on additional input and look forward to your response.

Thank you once again for your time and expertise.

Attachment Submitted filename: Response to editor.docx

# Peer review: Decision Letter 2

**DOI:** 10.1371/journal.pone.0356551.r005

**Authors:** Rizwan Jouhar

20 Jul 2025

Dear Dr. Demessie,

Thank you for submitting your manuscript to PLOS ONE. After careful consideration, we feel that it has merit but does not fully meet PLOS ONE’s publication criteria as it currently stands. Therefore, we invite you to submit a revised version of the manuscript that addresses the points raised during the review process.

Please submit your revised manuscript by Sep 03 2025 11:59PM. If you will need more time than this to complete your revisions, please reply to this message or contact the journal office at plosone@plos.org. When you're ready to submit your revision, log on to https://www.editorialmanager.com/pone/ and select the 'Submissions Needing Revision' folder to locate your manuscript file.

- A rebuttal letter that responds to each point raised by the academic editor and reviewer(s). You should upload this letter as a separate file labeled 'Response to Reviewers'.
- A marked-up copy of your manuscript that highlights changes made to the original version. You should upload this as a separate file labeled 'Revised Manuscript with Track Changes'.
- An unmarked version of your revised paper without tracked changes. You should upload this as a separate file labeled 'Manuscript'.

If applicable, we recommend that you deposit your laboratory protocols in protocols.io to enhance the reproducibility of your results. Protocols.io assigns your protocol its own identifier (DOI) so that it can be cited independently in the future. For instructions see: https://journals.plos.org/plosone/s/submission-guidelines#loc-laboratory-protocols. Additionally, PLOS ONE offers an option for publishing peer-reviewed Lab Protocol articles, which describe protocols hosted on protocols.io. Read more information on sharing protocols at https://plos.org/protocols?utm_medium=editorial-email&utm_source=authorletters&utm_campaign=protocols.

We look forward to receiving your revised manuscript.

Kind regards,

Rizwan Jouhar

Academic Editor

PLOS ONE

Journal Requirements:

1.If the reviewer comments include a recommendation to cite specific previously published works, please review and evaluate these publications to determine whether they are relevant and should be cited. There is no requirement to cite these works unless the editor has indicated otherwise.

2.Please review your reference list to ensure that it is complete and correct. If you have cited papers that have been retracted, please include the rationale for doing so in the manuscript text, or remove these references and replace them with relevant current references. Any changes to the reference list should be mentioned in the rebuttal letter that accompanies your revised manuscript. If you need to cite a retracted article, indicate the article’s retracted status in the References list and also include a citation and full reference for the retraction notice.

Additional Editor Comments:

I appreciate the authors’ thorough and thoughtful revisions. Minor revisions are suggested by one reviewer to further strengthen the study.

Reviewer 2 Comments

"The authors have revised the manuscript based on my suggestions from the previous peer review. However, there are still some areas that need to be revised, but I forgot to point out the significant figures of the AORs and their 95% CIs in the previous peer review.

Please refer to the following authoritative source:

https://www.cochrane.org/authors/handbooks-and-manuals/style-manual/numbers-statistics-and-units/statistical-and-mathematical-presentation

The significant figures of the odds ratios and their 95% CIs should be two decimal places.

Please correct the significant figures in the following parts:

In the text:

L35-38

L443-446

L450

and

Table 3"

[Note: HTML markup is below. Please do not edit.]

Reviewers' comments:

Reviewer's Responses to Questions

**Comments to the Author**

Reviewer #1: All comments have been addressed

Reviewer #4: All comments have been addressed

**********

2. Is the manuscript technically sound, and do the data support the conclusions?

Reviewer #1: Yes

Reviewer #4: Partly

**********

3. Has the statistical analysis been performed appropriately and rigorously?

Reviewer #1: Yes

Reviewer #4: Yes

**********

4. Have the authors made all data underlying the findings in their manuscript fully available?

The PLOS Data policy

Reviewer #1: Yes

Reviewer #4: Yes

**********

5. Is the manuscript presented in an intelligible fashion and written in standard English?

Reviewer #1: Yes

Reviewer #4: Yes

**********

Reviewer #1: Thank you for the opportunity to review the revised manuscript titled, "Implementation of Objective Structured Clinical Examination among Midwifery and Nursing Educators at South and Central Region Universities in Ethiopia, 2023." The authors have addressed all previous comments thoroughly and satisfactorily.

Overall, the revised manuscript demonstrates considerable improvement and meets the standards expected by Journal. The authors have successfully addressed all concerns raised by reviewers, and their comprehensive and rigorous revisions substantially enhance the manuscript’s quality and impact.

Reviewer #4: The authors have revised the manuscript based on my suggestions from the previous peer review. However, there are still some areas that need to be revised, but I forgot to point out the significant figures of the AORs and their 95% CIs in the previous peer review.

Please refer to the following authoritative source:

https://www.cochrane.org/authors/handbooks-and-manuals/style-manual/numbers-statistics-and-units/statistical-and-mathematical-presentation

The significant figures of the odds ratios and their 95% CIs should be two decimal places.

Please correct the significant figures in the following parts:

In the text:

L35-38

L443-446

L450

and

Table 3

**********

what does this mean?). If published, this will include your full peer review and any attached files.

If you choose “no”, your identity will remain anonymous but your review may still be made public.

**Do you want your identity to be public for this peer review?**  For information about this choice, including consent withdrawal, please see our Privacy Policy

Reviewer #1: **Yes:** Hye Won Jeong

Reviewer #4: **Yes:** Ryuichiro ARAKI

**********

[NOTE: If reviewer comments were submitted as an attachment file, they will be attached to this email and accessible via the submission site. Please log into your account, locate the manuscript record, and check for the action link "View Attachments". If this link does not appear, there are no attachment files.]

While revising your submission, please upload your figure files to the Preflight Analysis and Conversion Engine (PACE) digital diagnostic tool, https://pacev2.apexcovantage.com/. PACE helps ensure that figures meet PLOS requirements. To use PACE, you must first register as a user. Registration is free. Then, login and navigate to the UPLOAD tab, where you will find detailed instructions on how to use the tool. If you encounter any issues or have any questions when using PACE, please email PLOS at figures@plos.org

# Author comment: Author response to Decision Letter 3

**DOI:** 10.1371/journal.pone.0356551.r006

22 Jul 2025

Response to Reviewer 2

Thank you for your valuable feedback and comments on our manuscript. We have carefully reviewed your suggestions and have made the necessary corrections, including the significant figures of the adjusted odds ratios (AORs) and their 95% confidence intervals (CIs) to two decimal places, as you recommended.

The revisions have been incorporated in the following sections:

• In the text: Lines 35-38, 443-446, and 450

• Table 3

� We appreciate your thorough review and guidance, which have greatly improved the quality of our manuscript. Thank you once again for your support.

Dear Reviewers and Editors,

We sincerely appreciate your constructive feedback, which has been invaluable in helping us refine and improve the manuscript. Your support and guidance have significantly enhanced the overall quality of our work. We are happy to incorporate any further adjustments based on your additional input and look forward to hearing from you.

Attachment Submitted filename: Response to Reviewer 2.docx

# Peer review: Decision Letter 3

**DOI:** 10.1371/journal.pone.0356551.r007

**Authors:** Hamid Reza Baradaran

27 Jan 2026

Dear Dr. Demessie,

Thank you for submitting your manuscript to PLOS ONE. After careful consideration, we feel that it has merit but does not fully meet PLOS ONE’s publication criteria as it currently stands. Therefore, we invite you to submit a revised version of the manuscript that addresses the points raised during the review process.

Please submit your revised manuscript by Mar 13 2026 11:59PM. If you will need more time than this to complete your revisions, please reply to this message or contact the journal office at plosone@plos.org. When you're ready to submit your revision, log on to https://www.editorialmanager.com/pone/ and select the 'Submissions Needing Revision' folder to locate your manuscript file.

- A letter that responds to each point raised by the academic editor and reviewer(s). You should upload this letter as a separate file labeled 'Response to Reviewers'.
- A marked-up copy of your manuscript that highlights changes made to the original version. You should upload this as a separate file labeled 'Revised Manuscript with Track Changes'.
- An unmarked version of your revised paper without tracked changes. You should upload this as a separate file labeled 'Manuscript'.

If applicable, we recommend that you deposit your laboratory protocols in protocols.io to enhance the reproducibility of your results. Protocols.io assigns your protocol its own identifier (DOI) so that it can be cited independently in the future. For instructions see: https://journals.plos.org/plosone/s/submission-guidelines#loc-laboratory-protocols. Additionally, PLOS ONE offers an option for publishing peer-reviewed Lab Protocol articles, which describe protocols hosted on protocols.io. Read more information on sharing protocols at https://plos.org/protocols?utm_medium=editorial-email&utm_source=authorletters&utm_campaign=protocols.

We look forward to receiving your revised manuscript.

Kind regards,

Hamid Reza Baradaran, M.D., Ph.D.,

Academic Editor

PLOS One

Journal Requirements:

If the reviewer comments include a recommendation to cite specific previously published works, please review and evaluate these publications to determine whether they are relevant and should be cited. There is no requirement to cite these works unless the editor has indicated otherwise.

Please review your reference list to ensure that it is complete and correct. If you have cited papers that have been retracted, please include the rationale for doing so in the manuscript text, or remove these references and replace them with relevant current references. Any changes to the reference list should be mentioned in the rebuttal letter that accompanies your revised manuscript. If you need to cite a retracted article, indicate the article’s retracted status in the References list and also include a citation and full reference for the retraction notice.

Additional Editor Comments:

Please explain more about measuring the implementation

[Note: HTML markup is below. Please do not edit.]

Reviewers' comments:

Reviewer's Responses to Questions

**Comments to the Author**

Reviewer #4: All comments have been addressed

Reviewer #5: (No Response)

Reviewer #6: All comments have been addressed

**********

2. Is the manuscript technically sound, and do the data support the conclusions?

Reviewer #4: Yes

Reviewer #5: Partly

Reviewer #6: No

**********

3. Has the statistical analysis been performed appropriately and rigorously?

Reviewer #4: Yes

Reviewer #5: No

Reviewer #6: No

**********

4. Have the authors made all data underlying the findings in their manuscript fully available?

The PLOS Data policy

Reviewer #4: Yes

Reviewer #5: No

Reviewer #6: No

**********

5. Is the manuscript presented in an intelligible fashion and written in standard English?

Reviewer #4: Yes

Reviewer #5: No

Reviewer #6: No

**********

Reviewer #4: (No Response)

Reviewer #5: Dear Authors

Thank you for your valuable study. I will provide you with some comments for improving your quality of reporting.

1- Why have you used OSCE for educators? Please explain the research gaps in detail.

2- You mentioned the study was cross- sectional; so, why did you use pretest?

3- If you gather quantitative and qualitative data, your study is mix-method.

4- What kinds of quantitative methods are used in the present study? Please mention it in the abstract section.

5- How did you assess and score the OSCE implementation? Please explain it in detail.

6- The introduction is too long; please summarize it in 3 short paragraphs: what’s the matter? General results of previous studies; research gaps and the present study objective/s.

7- How did you select participants in the selected universities?

8- Please add the qualitative questions, the methods of analysis, and the results of this part of study.

9- How did you assess the psychometric features of the tool using?

10- How did you AOR for five-point scale?

Be successful.

Reviewer #6: Dear author,

Thank you for submitting your manuscript to PLOS ONE journal.

Some comments for your manuscript:

Select keywords from the MeSH.

Write a brief introduction and remove unnecessary content.

Please refer to the author's guideline for editing the manuscript.

Is this a mixed methods study?

The study design is not clear. The research design could be a little more detailed and linked to a philosophical position in line with the aim of the study.

Improve your tables with academic formatting.

The results section contained some interesting but rather critical quotes. These need to be written in the accepted convention.

What has been done to ensure rigor in this study? Please explain more.

Discuss more about limitations of the study.

Make sure the referencing structure conforms to the convention of the journal.

Best Regards,

**********

what does this mean?). If published, this will include your full peer review and any attached files.

If you choose “no”, your identity will remain anonymous but your review may still be made public.

**Do you want your identity to be public for this peer review?**  For information about this choice, including consent withdrawal, please see our Privacy Policy

Reviewer #4: **Yes:** Ryuichiro ARAKI

Reviewer #5: No

Reviewer #6: No

**********

[NOTE: If reviewer comments were submitted as an attachment file, they will be attached to this email and accessible via the submission site. Please log into your account, locate the manuscript record, and check for the action link "View Attachments". If this link does not appear, there are no attachment files.]

To ensure your figures meet our technical requirements, please review our figure guidelines: https://journals.plos.org/plosone/s/figures

You may also use PLOS’s free figure tool, NAAS, to help you prepare publication quality figures: https://journals.plos.org/plosone/s/figures#loc-tools-for-figure-preparation.

NAAS will assess whether your figures meet our technical requirements by comparing each figure against our figure specifications.

# Author comment: Author response to Decision Letter 4

**DOI:** 10.1371/journal.pone.0356551.r008

20 Feb 2026

Thank you for your valuable feedback and comments on our manuscript. We have carefully reviewed your suggestions and have made the necessary corrections and attached as file specifically " response to reviewers".

Attachment Submitted filename: Point by point response to Reviewer#6.docx

# Peer review: Decision Letter 4

**DOI:** 10.1371/journal.pone.0356551.r009

**Authors:** Ayse Hilal Bati

1 Jul 2026

Dear Zerihun Demessie,

Thank you for submitting your manuscript to PLOS ONE. After careful consideration, we feel that it has merit but does not fully meet PLOS ONE’s publication criteria as it currently stands. Therefore, we invite you to submit a revised version of the manuscript that addresses the points raised during the review process.

Your research examines how effectively faculty members in nursing and midwifery departments at different universities in Ethiopia design, manage, and evaluate OSCEs (Open Clinical Competencies Evaluation) in their teaching practices. In this sense, it answers many questions that lead you to your research objective. Your research report has been made more effective at different editing stages. However, it would be better if the differences in practice were clearly shown according to the universities, the academic ranks of the faculty members, and their experience levels. Also, how the training of clinical competencies assessed by OSCE is conducted in different faculties needs to be defined as an important component, as it is a very important determinant. Adding these details to your research report would make it more successful.

Please submit your revised manuscript by Aug 15 2026 11:59PM. If you will need more time than this to complete your revisions, please reply to this message or contact the journal office at plosone@plos.org. When you're ready to submit your revision, log on to https://www.editorialmanager.com/pone/ and select the 'Submissions Needing Revision' folder to locate your manuscript file.

- A letter that responds to each point raised by the academic editor and reviewer(s). You should upload this letter as a separate file labeled 'Response to Reviewers'.
- A marked-up copy of your manuscript that highlights changes made to the original version. You should upload this as a separate file labeled 'Revised Manuscript with Track Changes'.
- An unmarked version of your revised paper without tracked changes. You should upload this as a separate file labeled 'Manuscript'.

If you would like to make changes to your financial disclosure, please include your updated statement in your cover letter. Guidelines for resubmitting your figure files are available below the reviewer comments at the end of this letter.

If applicable, we recommend that you deposit your laboratory protocols in protocols.io to enhance the reproducibility of your results. Protocols.io assigns your protocol its own identifier (DOI) so that it can be cited independently in the future. For instructions see: https://journals.plos.org/plosone/s/submission-guidelines#loc-laboratory-protocols. Additionally, PLOS ONE offers an option for publishing peer-reviewed Lab Protocol articles, which describe protocols hosted on protocols.io. Read more information on sharing protocols at https://plos.org/protocols?utm_medium=editorial-email&utm_source=authorletters&utm_campaign=protocols.

As the corresponding author, your ORCID iD is verified in the submission system and will appear in the published article. PLOS supports the use of ORCID, and we encourage all coauthors to register for an ORCID iD and use it as well. Please encourage your coauthors to verify their ORCID iD within the submission system before final acceptance, as unverified ORCID iDs will not appear in the published article. *Only*  the individual author can complete the verification step; PLOS staff *cannot*  verify ORCID iDs on behalf of authors.

We look forward to receiving your revised manuscript.

Kind regards,

Ayse Hilal Bati, Professor

Academic Editor

PLOS One

Journal Requirements:

If the reviewer comments include a recommendation to cite specific previously published works, please review and evaluate these publications to determine whether they are relevant and should be cited. There is no requirement to cite these works unless the editor has indicated otherwise.

Please review your reference list to ensure that it is complete and correct. If you have cited papers that have been retracted, please include the rationale for doing so in the manuscript text, or remove these references and replace them with relevant current references. Any changes to the reference list should be mentioned in the rebuttal letter that accompanies your revised manuscript. If you need to cite a retracted article, indicate the article’s retracted status in the References list and also include a citation and full reference for the retraction notice.

Additional Editor Comments (if provided):

Dear Zerihun Demessie,

Your research examines how effectively faculty members in nursing and midwifery departments at different universities in Ethiopia design, manage, and evaluate OSCEs (Occupational Health and Safety Evaluations) in their teaching practices. In this sense, it answers many questions that lead you to your research objective.

Your research report has been improved in various stages of editing. However, it would be better if the differences in implementation were clearly shown according to the universities, the academic ranks of the faculty members, and their experience levels. Furthermore, how the training for clinical competencies assessed by OSCEs is conducted in different faculties needs to be defined as an important component, as it is a crucial determinant.

Adding these details to your research report would make it even more successful.

[Note: HTML markup is below. Please do not edit.]

Reviewers' comments:

Reviewer's Responses to Questions

**Comments to the Author**

Reviewer #4: All comments have been addressed

**********

2. Is the manuscript technically sound, and do the data support the conclusions?

Reviewer #4: Yes

**********

3. Has the statistical analysis been performed appropriately and rigorously?

Reviewer #4: Yes

**********

4. Have the authors made all data underlying the findings in their manuscript fully available?

The PLOS Data policy

Reviewer #4: Yes

**********

5. Is the manuscript presented in an intelligible fashion and written in standard English?

Reviewer #4: Yes

**********

Reviewer #4: All previously raised concerns have been adequately addressed, and the paper is now acceptable for publication.

**********

what does this mean?). If published, this will include your full peer review and any attached files.

If you choose “no”, your identity will remain anonymous but your review may still be made public.

**Do you want your identity to be public for this peer review?**  For information about this choice, including consent withdrawal, please see our Privacy Policy

Reviewer #4: **Yes:** Ryuichiro ARAKI

**********

[NOTE: If reviewer comments were submitted as an attachment file, they will be attached to this email and accessible via the submission site. Please log into your account, locate the manuscript record, and check for the action link "View Attachments". If this link does not appear, there are no attachment files.]

To ensure your figures meet our technical requirements, please review our figure guidelines: https://journals.plos.org/plosone/s/figures

You may also use PLOS’s free figure tool, NAAS, to help you prepare publication quality figures: https://journals.plos.org/plosone/s/figures#loc-tools-for-figure-preparation.

NAAS will assess whether your figures meet our technical requirements by comparing each figure against our figure specifications.

# Author comment: Author response to Decision Letter 5

**DOI:** 10.1371/journal.pone.0356551.r010

17 Jul 2026

Subject: Response to academic editor

Dear Ayse Hilal Bati, Professor, Academic Editor, PLOS ONE

Thank you very much for your insightful and constructive feedback on our manuscript. We appreciate your recognition that the research report has become more effective through the editing stages and that it successfully addresses the core research objectives.

We have carefully reviewed your suggestions and have fully revised the manuscript to incorporate these vital details. a detailed summary of how these specific findings have been incorporated into the revised manuscript.

Thank you once again for your guidance in improving this work.

Attachment Submitted filename: Response to Reviwers.docx

# Editor report: Decision Letter 5

**DOI:** 10.1371/journal.pone.0356551.r011

**Authors:** Ayse Hilal Bati

29 Jul 2026

Dear Dr. Demessie,

Thank you for submitting your manuscript to PLOS ONE. After careful consideration, we feel that it has merit but does not fully meet PLOS ONE’s publication criteria as it currently stands. Therefore, we invite you to submit a revised version of the manuscript that addresses the points raised during the review process.

Please submit your revised manuscript by Sep 12 2026 11:59PM. If you will need more time than this to complete your revisions, please reply to this message or contact the journal office at plosone@plos.org. When you're ready to submit your revision, log on to https://www.editorialmanager.com/pone/ and select the 'Submissions Needing Revision' folder to locate your manuscript file.

- A letter that responds to each point raised by the academic editor and reviewer(s). You should upload this letter as a separate file labeled 'Response to Reviewers'.
- A marked-up copy of your manuscript that highlights changes made to the original version. You should upload this as a separate file labeled 'Revised Manuscript with Track Changes'.
- An unmarked version of your revised paper without tracked changes. You should upload this as a separate file labeled 'Manuscript'.

If you would like to make changes to your financial disclosure, please include your updated statement in your cover letter. Guidelines for resubmitting your figure files are available below the reviewer comments at the end of this letter.

If applicable, we recommend that you deposit your laboratory protocols in protocols.io to enhance the reproducibility of your results. Protocols.io assigns your protocol its own identifier (DOI) so that it can be cited independently in the future. For instructions see: https://journals.plos.org/plosone/s/submission-guidelines#loc-laboratory-protocols. Additionally, PLOS ONE offers an option for publishing peer-reviewed Lab Protocol articles, which describe protocols hosted on protocols.io. Read more information on sharing protocols at https://plos.org/protocols?utm_medium=editorial-email&utm_source=authorletters&utm_campaign=protocols.

As the corresponding author, your ORCID iD is verified in the submission system and will appear in the published article. PLOS supports the use of ORCID, and we encourage all coauthors to register for an ORCID iD and use it as well. Please encourage your coauthors to verify their ORCID iD within the submission system before final acceptance, as unverified ORCID iDs will not appear in the published article. *Only*  the individual author can complete the verification step; PLOS staff *cannot*  verify ORCID iDs on behalf of authors.

We look forward to receiving your revised manuscript.

Kind regards,

Ayse Hilal Bati, Professor

Academic Editor

PLOS One

Journal Requirements:

If the reviewer comments include a recommendation to cite specific previously published works, please review and evaluate these publications to determine whether they are relevant and should be cited. There is no requirement to cite these works unless the editor has indicated otherwise.

Please review your reference list to ensure that it is complete and correct. If you have cited papers that have been retracted, please include the rationale for doing so in the manuscript text, or remove these references and replace them with relevant current references. Any changes to the reference list should be mentioned in the rebuttal letter that accompanies your revised manuscript. If you need to cite a retracted article, indicate the article’s retracted status in the References list and also include a citation and full reference for the retraction notice.

Additional Editor Comments:

Dear Authors,

I am pleased that you have taken my suggestions into account and made revisions. However, I did not see any improvements, particularly in the discussion section, regarding the interpretation of the data you obtained. I regret to notice that this section, which shows how the research results can be interpreted and applied to further development, is missing from the article. If this correction is also made, the data will gain meaning and the article will be ready for publication in PLOS One.

Thank you.

[Note: HTML markup is below. Please do not edit.]

To ensure your figures meet our technical requirements, please review our figure guidelines: https://journals.plos.org/plosone/s/figures

You may also use PLOS’s free figure tool, NAAS, to help you prepare publication quality figures: https://journals.plos.org/plosone/s/figures#loc-tools-for-figure-preparation.

NAAS will assess whether your figures meet our technical requirements by comparing each figure against our figure specifications.

# Author comment: Author response to Decision Letter 6

**DOI:** 10.1371/journal.pone.0356551.r012

31 Jul 2026

Response to Academic Editor– [PONE-D-24-18847R4]

Dear Ayse Hilal Bati, Professor, Academic Editor, PLOS ONE

Thank you for your follow-up feedback on our manuscript: [PONE-D-24-18847R4]). We sincerely appreciate your guidance regarding the need for a clearer, actionable interpretation of our findings in the Discussion section.

In line with your suggestion, we have thoroughly revised this section to provide a clear interpretation of our findings, rather than simply restating the results. Specifically, we have incorporated possible explanations for the observed variations in OSCE implementation across departments, universities, and faculty experience/rank, supported by relevant literature. We have also added a recommendation encouraging future researchers to conduct comparative studies, as such comparisons were beyond the scope of our study objectives.

All new additions have been highlighted using [Track Changes] in the revised manuscript.

We believe these revisions have strengthened the manuscript and given the data greater meaning and applicability, as you kindly noted. We are grateful for your guidance throughout this process and for your consideration of our work for publication in PLOS ONE.

Please let us know if any further revisions are required.

Thank you once again for your time and valuable input.

Sincerely,

Zerihun Demessie / Corresponding Author

Department of Midwifery, Hossana College of Health Science, Hossana, Ethiopia

Attachment Submitted filename: Response to Academic Editor.docx

# Editor report: Decision Letter 6

**DOI:** 10.1371/journal.pone.0356551.r013

**Authors:** Ayse Hilal Bati

4 Aug 2026

Implementation of Objective Structured Clinical Examination among Midwifery and Nursing Educators at South and Central Region Universities in Ethiopia, 2023

PONE-D-24-18847R6

Dear Dr. Zerihun Demessie,

We’re pleased to inform you that your manuscript has been judged scientifically suitable for publication and will be formally accepted for publication once it meets all outstanding technical requirements.

Within one week, you’ll receive an e-mail detailing the required amendments. When these have been addressed, you’ll receive a formal acceptance letter and your manuscript will be scheduled for publication.

An invoice will be generated when your article is formally accepted. Please note, if your institution has a publishing partnership with PLOS and your article meets the relevant criteria, all or part of your publication costs will be covered. Please make sure your user information is up-to-date by logging into Editorial Manager at Editorial Manager® and clicking the ‘Update My Information' link at the top of the page. For questions related to billing, please contact billing support.

If your institution or institutions have a press office, please notify them about your upcoming paper to help maximize its impact. If they’ll be preparing press materials, please inform our press team as soon as possible -- no later than 48 hours after receiving the formal acceptance. Your manuscript will remain under strict press embargo until 2 pm Eastern Time on the date of publication. For more information, please contact onepress@plos.org.

Kind regards,

Ayse Hilal Bati, Professor

Academic Editor

PLOS One

Additional Editor Comments (optional):

Dear Authors,

I appreciate your efforts to improve your work based on these suggestions. Your statement that you will use the suggestions regarding the research design in your future studies demonstrates your enthusiasm for the work. Thank you very much for your research and effort.

Reviewers' comments:

# Editor report: Acceptance letter

**DOI:** 10.1371/journal.pone.0356551.r014

**Authors:** Ayse Hilal Bati

PONE-D-24-18847R6

PLOS One

Dear Dr. Dilebo,

I'm pleased to inform you that your manuscript has been deemed suitable for publication in PLOS One. Congratulations! Your manuscript is now being handed over to our production team.

At this stage, our production department will prepare your paper for publication. This includes ensuring the following:

* All references, tables, and figures are properly cited

* All relevant supporting information is included in the manuscript submission,

* There are no issues that prevent the paper from being properly typeset

You will receive further instructions from the production team, including instructions on how to review your proof when it is ready. Please keep in mind that we are working through a large volume of accepted articles, so please give us a few days to review your paper and let you know the next and final steps.

Lastly, if your institution or institutions have a press office, please let them know about your upcoming paper now to help maximize its impact. If they'll be preparing press materials, please inform our press team within the next 48 hours. Your manuscript will remain under strict press embargo until 2 pm Eastern Time on the date of publication. For more information, please contact onepress@plos.org.

You will receive an invoice from PLOS for your publication fee after your manuscript has reached the completed accept phase. If you receive an email requesting payment before acceptance or for any other service, this may be a phishing scheme. Learn how to identify phishing emails and protect your accounts at https://explore.plos.org/phishing.

If we can help with anything else, please email us at customercare@plos.org.

Thank you for submitting your work to PLOS One and supporting open access.

Kind regards,

PLOS ONE Editorial Office Staff

on behalf of

Dr. Ayse Hilal Bati

Academic Editor

PLOS One
